# The Nervous System of the Human Body; as Explained in a Series of Papers Read before the Royal Society of London. With an Appendix of Cases and Consultations on Nervous Diseases

**Published:** 1840-01

**Authors:** 


ART. V.

1. *The Nervous System of the Human Body; as explained in a Series of Papers read before the Royal Society of London. With an Appendix of Cases and Consultations on Nervous Diseases.* By Sir CHARLES BELL, K.G.H., F.R.SS.L. & E., &c. &c. Third Edition.—*Edinburgh*, 1836. 8vo, pp. 501. With Plates.

2. *Narrative of the Discoveries of Sir Charles Bell in the Nervous System.* By ALEXANDER SHAW, Assistant Surgeon to the Middlesex Hospital.—*London*, 1839. 8vo, pp. 232.

3. *Documents and Dates of Modern Discoveries in the Nervous System.* —*London*, 1839. 8vo, pp. 172.

FEW controversies are more bitter than those which relate to priority of discovery; few present more temptations to fraud and misrepresentation; none are more profitless to the world. We can excuse a man for not feeling satisfied as to the reception of his discovery. We can justify him for his efforts to induce a more correct appreciation of it; because in so doing he is labouring for others as well as for himself. But what, save a purely selfish motive, can induce him to endeavour to raise himself upon the reputation of another? The *knowledge* is that which the world wants; of what practical importance is it by whom that knowledge has been first attained?

We do not think, however, that the world should be left in ignorance on the subject; because, if the real claimants do not make known their pretensions, there will always be found upstarts quite ready to seize the reward, without the slightest title to it. It seems to us to be the duty and the best interest of a discoverer, to present to the public a simple history of his progress towards the truth; not disguising his early errors, or endeavouring to clothe with more precise form the vague ideas with which he started; but recording every fact and opinion as it presented itself, with a scrupulous attention to dates. If he do this faithfully, the very air of *vraisemblance* which his history will present, will carry conviction along with it; and he may safely leave it to the public to decide between his claims and those put forward by others. Further than this, we cannot think that he should go. Angry recriminations can serve no good purpose, and only tend to keep alive the worst feelings of our nature, besides affording strong temptations to violate truth and sincerity. Nothing in such a history carries more weight than the candid acknowledgment of assistance supplied or of corrections received from other sources. On the other hand, nothing tends to excite suspicion so much as the exclusive arrogation to oneself of the whole merit of the discovery; since it can seldom happen in a lengthened enquiry that the conductor of it has not been indebted for facts or suggestions to others.

We have been led into these remarks by certain late events of much interest in the history of physiological science; and more particularly by the fresh discussion which has recently commenced as to the respective claims of Sir Charles Bell, Mr. Mayo, and M. Magendie, to the discovery of the double character of the spinal nerves, and of the true functions of the fifth and seventh cranial nerves—unquestionably one of the greatest discoveries which the present age has witnessed. It is the object of the present article to examine the whole subject; and to award to every claimant what we consider his just right, without favour or affection. The works from which our materials will be derived are principally the three whose titles stand at the head of this article. That of Sir C. Bell appears to be a reprint of the Memoirs which he has presented at different times to the Royal Societies of London and Edinburgh; with an introduction, containing a general view of the functions of the nervous system, and an account of his own early enquiries in regard to them, as well as a history of the opinions previously entertained on the subject. We say *appears*, because we are bound to state that the most important of all these memoirs, in a historical point of view, has been so greatly modified, without due intimation of a change, as to be almost valueless for the purpose of reference. To this point we shall have to return hereafter. The history of the author's early enquiries is vague and unsatisfactory. It does not contain a single date. It omits all the errors into which the author was at first led, either by previously formed ideas, or by misinterpretation of observed facts. And it includes no acknowledgment of assistance or correction from others.

Now, it will be seen from our subsequent history that we entertain as high an opinion of Sir C. Bell's merits as a discoverer, as it would be easy to form by the examination of his successive publications. In fact, there is scarcely any point in the *later* history in which we do not coincide with his historian, Mr. A. Shaw, who, from his connection with him, might be suspected of partiality in his favour. But it is in the *early* history that both are deficient; and, as Mr. Shaw enters fully into this, for the purpose of exposing what he asserts to be the misrepresentations of others, the omission of the first errors of Sir C. Bell—in fact the positive contradiction of them—is a piece of seeming disingenuousness for which we cannot account, and which, if we are correct in our estimate of it, is in the highest degree discreditable to him. The remainder of Mr. Shaw's book is, on the whole, so candid and correct, that we should not have in the least suspected him of acting in this manner; and, if we had not come into possession of the document in question, we should have accepted his quotations from it as a fair statement of the opinions there expressed,—the fact really being, that the erroneous ones are kept back. Putting aside this point, we have little fault to find with the performance. Mr. Mayo is, we think, very justly reprobated for his attempts to strip Sir C. Bell of all the merit of priority, in favour of M. Magendie, when he could substantiate no claim for himself; and the mode in which he brought forward the corrections which he made upon Sir C. Bell's early views is shown in a very different light from that in which he has himself presented it. To these points we shall hereafter return; and we can here only express our regret that such differences have arisen between two individuals possessing high and independent claims to eminence.

The "Documents and Dates" are far from supplying a complete history of Neurology. Such a work, executed by one who should be at the same time impartial and well qualified, would be both interesting and useful. Regarding the impartiality of the present editor, we cannot say much. The collection is so evidently designed to exhibit the supposed anticipation of Sir C. Bell by Mr. Alexander Walker, that we cannot have much doubt as to the source whence it has originated. We shall hereafter have occasion to enquire minutely into this new claim, and also into the editor's qualifications for accurately representing the opinions of other writers. The chief value of the book is its containing *the whole* of the important document we just now referred to;—namely, the unpublished tract of Sir C. Bell, distributed among his friends in 1811. Our subsequent strictures are based upon the supposition of this being a correct reprint; and, from the comparisons we have been able to make, we have no reason to think it otherwise.

We entered at some length, on a former occasion, into a review of the present state of our knowledge regarding the physiology of the nervous system. This review, however, had a special bearing on one particular department—the functions of the spinal cord, which was the subject at that time under discussion. At present, we propose to take a more general and less detailed survey, in which we shall introduce some modifications of our former views, rendered necessary by late additions to our knowledge. Our purpose in so doing is to enable our readers, in following us through the subsequent history, to carry with them a clear view of what may be at present regarded as the highest generalizations on the subject, so as to be able to form a more correct appreciation of the contributions of each discoverer towards the science of Neurology. It is not a little remarkable, that most of what is definitely known regarding the special functions of the nervous system has been ascertained within the last thirty years. We shall find that many *speculations* had been previously offered in respect to them; but few of these could be regarded as more than vague and ill-supported hypotheses. Many correct observations of phenomena had been made, and sagacious inferences had been erected upon them; but all were wanting in precision, until the guiding principle was established, which combined and directed the efforts that were previously unstable and desultory.

The ancient notion of the operation of the nervous system in producing its diversified phenomena was, apparently, a very simple and comprehensive one, but was really of no value whatever. It consisted in regarding the central organ as a sort of gland in which "animal spirits" were elaborated, and the nervous trunks as the channels by which these were conveyed to all parts of the system for their respective purposes. It was very convenient, therefore, to refer every manifestation of nervous agency, which could not otherwise be explained, to the operation of the "animal spirits;" and, however absurd and unphilosophical this may appear, it must be remembered that many physiologists at the present time are in the habit of seeking in the "vital principle" a similar refuge from their difficulties. We shall perceive that the progress of neurological research has been manifested in the more complete separation and classification of its varied phenomena, and in the assignment of these to distinct parts of the nervous apparatus. In the same manner, then, the philosophic physiologist will seek to explain the other phenomena of life by the properties of the organs manifesting them; and these may or may not be analogous to those manifested in the inorganic world.

Our present knowledge of the functions of the nervous system enables us to view it under three different aspects:—1. As the instrument of the mind; by which it acquires a knowledge of the external world through the medium of sensation, and operates upon it by an exercise of volition. 2. As the means by which various movements are excited in the bodily structure, which are immediately necessary to the performance of the organic functions, and to its protection from injury. 3. As the means by which the organic functions themselves are harmonised and controlled, and influenced by emotional states of mind.

Now, we find in Vertebrata three evident divisions in the nervous apparatus, to which these classes of functions may be respectively assigned. These are, the brain and nerves proceeding from it, the (true) spinal cord and nerves proceeding from it, and the (so-called) sympathetic system of isolated ganglia and nerves. The nerves proceeding from the brain are generally united with those connected with the spinal cord, so that the distinction between them cannot be traced without considerable difficulty; but in many invertebrate animals the distinction between the nerves of the two systems is more easily demonstrated.

In each division of the nervous system two kinds of structure are evident: the one consisting of continuous fibres, the other of a plexus of blood-vessels in which the fibres appear lost, and of an apparently confused mass of granules. Of the first kind the nervous trunks are exclusively composed; and as these are known to be simply *conductors* of whatever changes take place in the central or peripheral organs, there is good reason to believe that the fibrous structure, wherever it exists, has the same function. The fibres are of two classes; those proceeding from the circumference to the centre, or *afferent* nerves; and those proceeding from the centre to the circumference, or *efferent* nerves. The fibres of both kinds seem to communicate at their central terminations with the vascular plexus and granular substance. This constitutes what is termed the *cortical* substance of the brain,—the *gray matter* of the spinal cord,—and the *nuclei* of the ganglia. The *afferent* fibres of the first two systems, which commence on the general surface or in special sensory organs, have a corresponding substance (though its elements are somewhat differently arranged) at their peripheral origin. It is evident, both in the retina, the expansion of the auditory and olfactory nerves, and in the papillæ of the skin and tongue, that the vascular system is there peculiarly brought into relation with the nervous fibres; and a granular structure is always present, which seems intermediate between them. The peripheral termination of the *afferent* nerves, however, is different; they form a plexus in the muscles upon which they act; and in this no free extremities are discoverable.

It is well known that the active influence of the vascular system is essential to the production of any changes in the nervous apparatus. If the circulation of blood through the brain be suspended for an instant, insensibility supervenes; that is to say, the connexion of the mind with the external world is suspended. The spinal system may still be excited to action. But if (as in syncope) the circulation through the spinal cord be also weakened, its power of producing motions in respondence to impressions is diminished in like proportion. In the same manner the production of impressions on the peripheral origins of the afferent nerves appears equally dependent upon the active influence of the vascular system. Every one knows that cold, which retards the circulation of blood through the skin, diminishes also its sensibility; and obstruction to the circulation by any other cause, such as pressure on the arterial trunks, produces the same effect. We have no opportunities of observing such affections of the special sensory organs, except in general syncope; and then, as the brain is also affected, there is no proof that their impressibility is diminished, though it can scarcely be doubted that this is the case. It is evident, therefore, that the changes which take place at the peripheral origins of the afferent nerves bear considerable analogy with those which occur at the central origins of the efferent; and the striking analogy of structure already pointed out would thus lead us to regard the gray matter of the central organs as the true seat of all the changes which take place in them. This belief is strengthened by various facts, of which some will be presently mentioned.

Less is known of the structure of the third or sympathetic system. Its fibres seem less perfect and distinct than those of the two former. It has been maintained by some that the gray matter is not confined to the nuclei of the ganglia, but is distributed through the trunks. The investigations of Remak* and others, however, appear to have shown that the gray colour is that of the fibres themselves, and that the vascular and granular structure is confined to the nuclei; so that there are, probably, afferent and efferent fibres in this system also, although they cannot be demonstrated by experiment.

1. The *cerebral*, or, as it has been termed, *sensori-volitional* system, bears a less proportion to the rest in the simpler tribes of animals than in the higher; and it cannot always be distinguished from that which corresponds with the spinal cord of Vertebrata, and which (for reasons to be presently given) we shall call the *excito-motor system*, or *system of reflexion.* Wherever the mouth is placed on a prominent part of the body, and is accompanied by organs of special sensation which determine the movement of the trunk in that direction, we find a ganglion, which appears to have a presiding or controlling influence over the rest; and this may be regarded as the chief, if not the only seat of sensibility, and as the originator of the voluntary movements: in other words, the instrument of whatever *mind* these animals can be regarded as possessing. In the radiated tribes, however, we find the mouth in the centre of the body, and the nervous system forming a ring around it; the ganglia on this ring (each of which, in the asterias, is connected with what seems to be an eye at the end of the corresponding ray) are evidently all equal in their endowments, and progression takes place indifferently in any direction.

Where, however, the cerebral ganglia are distinct, as in most of the Molluscous and Articulated tribes, we find that, however small they are in proportion to those of the excito-motor system, they send nerves to every part of the body supplied by the latter; for the purpose, it would seem, of controlling, harmonising, or antagonising its actions. These nerves proceed as connecting trunks from the cerebral ganglia to the other centres; and then divide into filaments, which unite with those proceeding from them

* See B. and F. M. R., Vol. VII., p. 501.

to the several organs. Each organ, therefore, receives four sets of fibres: an afferent and efferent set, which connect it with the cerebral ganglia, and are the channels of sensation and of the influence of the will; and an afferent and efferent set which connect it with its particular ganglion, and serve to convey the stimulus of impressions which produce motions by reflected influence.

In proportion as the special sensory organs are developed, and the animal less completely governed by *mere* instinct, we find the cerebral ganglia and system of nerves more predominant. This is especially the case in vertebrata. Here it is united more closely, however, with the excito-motor system (for the sake, perhaps, of the protection which the osseous structure is adapted to afford to both); but it is still distinguishable. The fibres which form the crura cerebri are continuous with the fibrous structure of the spinal cord, and form part of each root of the spinal nerves, by which they are distributed with those of the spine itself. Above, they may be traced to the cortical substance, or to the insulated tracts of gray matter. The medullary substance of the cerebrum seems to consist in part of afferent and efferent fibres, but principally of fibres which serve to unite the different parts of the cortical substance with each other, constituting the various transverse and longitudinal commissures. These fibres of communication are especially abundant in the human brain, to whose bulk they contribute in large proportion.

2. The *true spinal* or *excito-motor* system consists, in the Vertebrata, of a continuous ganglion with nerves proceeding from it, which unite, as just stated, with those derived from the cerebrum. Its ganglionic character is shown by the existence of gray matter in its centre, in which the nervous fibres are lost, just as in the cortical substance of the brain, and in the nuclei of the ganglia of the invertebrata. Both roots of the spinal nerves are connected with it;* and each is thus made up of fibres from the cerebral system, and of fibres from this spinal ganglion. The spinal cord, or prolonged ganglion, of the vertebrata, differs greatly in its functions in different parts. That portion which is termed the medulla oblongata is the centre of the respiratory movements, and of those of deglutition. The lower part is concerned in the reflex movements of the general locomotive apparàtus, and of the sphincters of the rectum and bladder, as well as in other actions. Now, in the invertebrate animals we find these different ganglionic centres more or less disjoined. In the Mollusca we have generally a pedal ganglion, a respiratory ganglion, a stomato-gastric ganglion, and sometimes a palleal ganglion for regulating the motions of the mantle, which are partly concerned in respiration and partly in progression. These are generally quite insulated from one another, although each is connected with the cerebral ganglion, as formerly mentioned; and as the connecting trunk from a more distant centre sometimes passes through or over a nearer one in its way to the head, an appearance of communication between these is occasionally presented where none really exists. In the Articu-

* This was pointed out first by Bellingeri, and afterwards by Mr. Grainger (B. and F. M. R., Vol. V., p. 496); and has been subsequently in part confirmed by Remak (Ibid Vol. VIII., p. 504).

lata, as in the vertebrata, there is a repetition of similar parts in the different segments; and there is, accordingly, a repetition of ganglionic centres and corresponding nerves. Thus the double chain of ganglia, which runs along the ventral aspect of the body, is evidently a repetition of the pedal ganglion of the mollusca, and corresponds with the lower part of the spinal cord in vertebrata.* The respiratory ganglia are repeated in like manner; and the stomato-gastric system often possesses three, or even five distinct centres. Some of these, however, appear to belong in part to the sympathetic system. In the vertebrata all these centres are fused into a continuous mass. The term *spinal system* is a convenient one, if it is understood to include the scattered analogues of this system in the invertebrate animals.

The functions of this spinal system may be stated to consist in the reception of external impressions, which, being conveyed by the afferent nerves to the central organs, excite respondent motions by an impulse transmitted to the muscles through the efferent trunks. The nature of these has been fully discussed on a former occasion;† and although we did not then admit that the phenomena justified the attribution of them to a system of nerves distinct from the cerebral, as we now accord with Dr. M. Hall in representing them, we have nothing to alter in the account we there gave of the general character of the phenomena. That *sensation* is generally produced by the impressions which excite reflex actions is fully explained by what has been stated of the joint distribution of the cerebral and spinal systems of nerves; and it appears to us more clear than ever that sensation does not participate in actions of this class, in the higher animals at least, though it usually accompanies them. In regard to the lower tribes, however, there is more obscurity; since there sometimes appears, as already stated, little or no difference in the endowments of the different ganglia.

Several different groups of muscular actions are performed by this system; and all of these are closely connected with the maintenance of life. We find, however, that, as we ascend the scale of animal creation, many of them are gradually subordinated to those of the sensori-volitional system; and the same thing may be observed in the advancing growth of the new-born human infant. Thus the prehension of food by the lips, or mandibles, seems to be an action as completely reflex in its character, in many of the lower tribes, as that of respiration; we know that it is so in the young of man and other mammifera; yet in their adult state it becomes a voluntary act. The movement of deglutition is always a reflex one, however, and cannot be produced by a direct effort of the will. In the lower tribes, again, in which respiration is a mere organic function, and is not subservient to speech and other voluntary actions, it seems to be little affected by the cerebral system; whilst in man, we know that it is much controlled and modified by the will. This is still more true of the locomotive organs, whose reflex actions in man are entirely guided by the will, and are, in fact, scarcely distinguishable; whilst, in many of the lower tribes, the will appears merely to control and direct them.

* See Dr. Carpenter on the Nervous System of Invertebrata, p. 58.

† See Review of Hall, Grainger, Mayo, &c., in Vol. V.

There is a class of movements in which both these systems appear to participate;—those, namely, which result from *emotions* of the mind, and which are particularly manifested in the respiratory apparatus. They would appear to originate in the cerebral system, since we know that *sensations* are necessary to excite these emotions; and there is reason to believe that, if the cerebral lobes be removed, no emotions can be excited. But they would seem conveyed rather by the spinal than by the cerebral nerves; for we find them retained, with the reflex actions of respiration, when the muscles are paralysed to voluntary influence; and if the respiratory nerves do not act in their usual manner, the power of expression is lost. The subject is at present involved in considerable obscurity.

As far as we are acquainted with the functions of the *cerebellum* (we eschew, for the present, the questions of *phrenology*), it would seem that this organ is concerned in regulating and controlling the movements of the body in such a manner as to maintain its equilibrium. From its connexions it would seem to belong partly to the cerebral and partly to the spinal system; and it is by no means impossible that it may, to a certain extent, regulate and combine the actions of both. It is generally found to be the largest in those animals which execute the greatest variety of movements; particularly if these be such as require a nice and continued equipoise of the body. Thus it is scarcely developed in fishes, whilst it is large in birds and mammalia. It is more highly developed in birds which hover long on the wing, as the kite, than in those which simply fly; and it is much larger in proportion to the spinal cord in man (who walks erect) than in any other of the mammalia; the semi-erect apes approaching nearest to him in this respect.*

3. The *sympathetic* or visceral system of nerves is more closely united with the spinal system in the lower classes than in the vertebrata. But even in the latter it is not entirely distinct. Late enquiries have shown that filaments derived from the cerebro-spinal nerves exist in those proceeding from the sympathetic ganglia; and that, on the other hand, fibres analagous to those of the sympathetic exist in the spinal nerves. The enquiries of Remak tend to prove that these fibres terminate in the ganglia, on the posterior roots of the spinal nerves, which may therefore be regarded as a part of the sympathetic system. It can scarcely be doubted, then, that these ganglia, and the fibres proceeding from them, have an influence on the nutritive processes; and this will account for the injury to these processes occasioned by section of the cerebro-spinal nerves, which have been usually regarded as having a less direct connexion with them.† Regarding the general functions of the sympathetic system, we have nothing to add to what we stated on a former occasion. It seems to us to possess the same kind of concern in the organic sympathies as the spinal system has in the evident sympathetic movements; and just as, in the latter case, muscular contraction may be excited in insulated parts without the influence of nervous agency, so do we believe

* See the Comparative Tables in Serres' Anatomie Comparée du Cerveau, tom. ii., pp. 173 and 423.

† We now partly agree, therefore, with Dr. M. Hall, in our view of the functions of these ganglia; though, for the reason formerly stated (Vol. V. p., 521), we cannot, with him, regard nutrition as *dependent* on them.

that the individual acts of nutrition and secretion are independent of the sympathetic system, although harmonised and controlled by it. To use the felicitous illustration of Dr. J. Reid, "the movements of a horse are independent of the rider on his back,—in other words, the rider does not furnish the conditions necessary for the movements of the horse,—but every one knows how much these movements may be influenced by the hand and heel of the rider."*

If the preceding outline be regarded as giving a fair summary of the present state of our knowledge on the subject, it will be evident, from a slight retrospective glance at the mode in which it has emerged from its original chaos, that three principal eras may be recognized in the history of its erection. The *first* is that of the separation of the sympathetic from the cerebro-spinal system. It is not easy to say who was the first to point out the anatomical distinctness of the two; but there can be no question that to Bichat we owe the first definite assignment of their respective functions. We believe him to have taught very erroneous opinions as to the nature of the influence of the sympathetic system on the processes of nutrition and secretion; yet the term which he applied to it—the *nervous system of organic life*,—in contradistinction to the cerebro-spinal system, which he designated the *nervous system of animal life*, is by no means inappropriate. We shall return to his opinions hereafter. The *second* era is that of the discovery by Bell of the structural distinctness of the *afferent* and *efferent* fibres of the cerebro-spinal system. The difference in their functions had long been noticed, and had by some been attributed to structural distinctness; but other physiologists of the highest eminence denied this, and no certainty could be said to exist on the subject. By his separation of the respiratory series from the general system of cerebro-spinal nerves, he may be regarded as having approached the *third* great improvement, which consisted in the separation of the *true spinal system*, of excitor and motor nerves, from the cerebral system of sensation and volition. This is principally the work of Dr. M. Hall. Previous physiologists (as we formerly pointed out) had observed insulated phenomena, which they attributed to the independent action of the spinal cord; and some had expressed the opinion that neither sensation nor volition were concerned in their production. By classifying and grouping together these phenomena, and combining them with many others first observed by himself, Dr. H. was able to specify in a much more full and precise manner than had ever before been done, the particular functions of the spinal cord, as distinguished from those of the brain. This was his first step; and he may be regarded as then on a par with those who had *functionally* distinguished the afferent from the efferent nerves. But many of his experiments and observations led him to the belief that the spinal and cerebral nerves are really as distinct from each other as the afferent and efferent, although, like them, generally bound together in the same trunk. This view, however, was not received by many of those physiologists who accorded with him in his earlier conclusions, being regarded by them as unsupported by anatomical evidence. To his own mind this evidence did not appear necessary; the *functional* difference being with him suffi-

* On the Functions of the Eighth Pair. Edinb. Med. and Surg. Journ., vol. 51.

cient to prove the structural distinctness. But others were not so readily convinced; and this is not surprising, when it is remembered that the immortal Haller positively denied the structural distinctness of the motor and sensory fibres, although he admitted that their influence was conducted in opposite directions. The anatomical evidence was supplied with regard to the Vertebrata by Mr. Grainger, and with regard to the Invertebrata by Dr. Carpenter; both of whom acted on hints thrown out by Dr. M. Hall, in bringing facts, some of which were new, and some previously known, to bear upon his doctrines.

We have traced the mode in which Dr. Hall arrived at this discovery, and we have seen that he advanced from *general* to *particular* facts. Now, it is a little curious that, if Sir C. Bell's attention had been less exclusively directed to the respiratory system, he would probably have arrived at the same conclusion by advancing from *particular* to *general* facts. If he had noticed that, in the invertebrate animals, not only the respiratory apparatus has a centre of nervous action distinct from the cerebral system, but that the stomato-gastric and locomotive nerves, and sometimes others, have equally distinct centres,—and that all these are combined in the spinal cord of the vertebrata,—he could scarcely have avoided the inference that their actions are of the same character, though usually more under the control of the cerebral system. It is by his having pointed out this fact that Dr. Carpenter appears to us to have contributed evidence in support of Dr. M. Hall's views, beyond what the mere proof of their applicability to these classes of animals would have afforded. Sir C. Bell had in many instances spoken of the respiratory nerves as not only functionally but structurally distinct from those of general motor power, even when bound up in the same sheath with them; and has pointed out that one may be paralysed without the other. To those who received his doctrines regarding the respiratory system, therefore, the extension of their views to Dr. M. Hall's excito-motor system need not be difficult.

We are now prepared to enter into a more detailed examination of the general history of neurological science; in this survey we shall notice but briefly the subject of our former enquiry, directing our attention rather to those topics which concern the discoveries of Sir C..Bell, Mayo, and Magendie. We would preface our summary with the observation which we formerly made on a similar occasion: "The claims to *discovery*, on a question like the present, are very difficult to adjust, if all the vague thoughts which have preceded them respecting the subject they involve are allowed to be brought in evidence of previous right;—thoughts, too, which would perhaps have been forgotten, if the clear statements of the writer whose claims to originality are opposed had not revived them." After *any* grand discovery has been made, it is generally easy to find some glimpses of it in the writings of previous philosophers. But we never think of giving to *them* the credit of it, unless they have contributed something more than a *guess* that such things *might possibly be*. Who, for example, ever thinks of depriving Watt of the merit of inventing the steam-engine, because the Marquis of Worcester, Savery, or Newcomen had partially and imperfectly employed the same motive power, but on principles very different? And yet Watt's *first* engine was but an improvement on Newcomen's.

If he had then stopped, and any other person had carried forward the improvement on principles which he there applied, but whose full development did not occur to him, it is evident that the merit would have been divided But, with that remarkable sagacity which characterised him, he was enabled to *lead* for many years the course of improvement, and to bring his invention to all that perfection which *theory* could give, without receiving important assistance from any one. Yet, in perfecting his machine, he did make use of the inventions of others; as the introduction of the crank, the parallel motion, and other contrivances, fully testify. But these improvements had no relation to his principle; they merely enabled him to carry it into more advantageous operation.

Now we think it will appear that Sir C. Bell stands very much in this position. In his earliest statement on the subject he developes a principle of enquiry which had not previously been made the subject of investigation. It cannot be questioned that he was at that time far from seeing the full bearing of this principle; and that it gradually acquired importance in his mind as new applications of it suggested themselves to him. But it is to be borne in mind that these applications were made by himself; and that the progressive modifications which his views have undergone have resulted, in almost every instance, from his own researches. In some points it will appear that he was anticipated by others; but we shall find strong reason to believe that he was ignorant of their results; and that, if he had become acquainted with them, he would have been earlier led to the development of truth, without his merit as a discoverer being in the least degree diminished.* In the solitary instance in which an important correction was supplied by another enquirer, that correction was derived from experiments made without regard to his principle, and by an individual who was *at that time* strenuously opposing it. If Sir C. Bell had relinquished the investigation before the publication of his first memoir in 1821, it would perhaps be rather difficult to say what precise claim to discovery he could have set up: since, as we shall presently see, a difference of function in the two roots of the spinal nerves had been previously guessed at; and, although he was the first to attempt an experimental solution of the question, he had arrived at no very definite results. But we know, through subsequent publications, not only of his own but of the late Mr. John Shaw's, that he was engaged in following out this enquiry during the ten years which followed the first statement of his views; and we are enabled to gather from them the degree of perfection they had attained, long before his first enunciation of them to the world. And we can trace in Mr. J. Shaw's papers their gradual modification and maturation, even better than in his own memoirs; for, as we shall hereafter see, the idea of a separate respiratory system early occurred to him; and in following it out, he would seem, taking *his own* publications alone, to have somewhat lost sight of the original subject of his enquiries. We shall presently attempt to develope what appears to have been the

* We here principally refer to the data with which the anatomy of the roots of the fifth pair might have supplied him. Much more was known on the subject, as we shall presently show, than he was aware of.

progress of his views in this important investigation; and we offer these preliminary remarks, lest it should be thought that, in bringing together the expressed opinions of previous authors, we are attempting to deprive him of the credit he so justly deserves. A historical survey of this kind has many points of interest, and some of practical utility. The former we need scarcely advert to; but the latter deserve a brief notice.

Many persons deprecate the study of the older authors on scientific subjects as a waste of time and mental energy. To a certain extent this is correct. To seek in them for that knowledge which they had no means of attaining, would be obviously absurd. No one expects to find a microscopical description of the tissues of the organized body before the era of the invention of the instrument. But it will often occur that men of original views suggest thoughts which they have no power of following out,—which are, in fact, in advance of their time; and these may be profitably taken up at a subsequent period. Moreover in those departments in which the phenomena are constantly presenting themselves to inspection, an acute observer will frequently seize almost intuitively the essential details, and transmit to posterity accounts of them which may be highly valuable as bases for further enquiries. Thus the descriptions of diseases, founded on symptoms alone, left us by Hippocrates, were probably not surpassed by those of any other physician down to the time of Sydenham; and the descriptions of various species of animals, including not merely their external form but their internal structure, which were drawn up under the direction of Aristotle, if not actually by him, would, if attended to by subsequent naturalists, have saved them from many errors, some of them egregious ones. We by no means recommend the study of by-gone authors to those who desire merely to acquaint themselves with the present state of the science they are pursuing; since to them it would be generally a misemployment of time. But on those who are pursuing the path of original enquiry in any department upon which the means of investigation were within reach of their predecessors, we would urge a careful research into their contributions, whether of fact or opinion, as a matter of interest as well as of duty. They will frequently thus be able to start from a more advanced position; they will often receive valuable assistance in their progress; and, when they have completed their work, they will be able to reply more successfully to the attacks of those (and some, we fear, will always be found to cavil thus) who represent their discoveries as "nothing new."

With these views, then, we shall commence our historical retrospect. We by no means assert that it is a complete survey of the literature of this interesting subject. Many names and opinions might, we doubt not, be added; but we feel confident in the correctness of our statements on all essential points, and in the truth of the view they present of the *general* state of knowledge at each epoch.

The connexion of the nervous system with the functions of sensation and motion has been known from the most ancient times; and it would be difficult, if not impossible, to trace the discoverer of this relation. It appears, too, that there was very early a vague idea that different parts of the structure might minister to these two functions respectively. Hierophilus and Erasistratus, the first dissectors of a human body, made a distinction between the Νεῦροι κινητικοὶ and the Νεῦροι αἰσθητικοὶ; but it does not appear that they gave any very definite statements as to the details of the subdivision. Aretæus and other ancient writers partook in this opinion, drawing strong evidence in its favour from the not unfrequent occurrence of loss of one function while the other remained unaffected. None of them, however, advanced so near to the truth as Galen; and to his writings, therefore, we may refer as giving the highest view of the state of knowledge among the ancients on this interesting subject. The following summary will, we believe, present a fair general statement of his opinions.

Galen certainly believed that the nerves of motion are *structurally* distinct from those of sensation, and that they are connected with different parts of the brain. He stated that the latter are all soft, and the former hard (which is pretty nearly true); that the latter arise from the anterior, and the former from the posterior part of the brain. It is on record that his attention was particularly directed to this subject by his having been called on by some of his contemporaries to account for the manner in which he had cured a partial paralysis of the finger, by applications made to the spine. In reply he told them that two sets of nerves went to every part; one to endow the skin or other organ with sensibility; the other to give the muscles a power of voluntary action. But he had no idea of any functional difference in the nervous trunks, beyond that arising from their texture. He imagined that the soft nerves were more susceptible of impressions, and the hard ones less impressible, but stronger, and therefore better fitted for action. He maintained, too, that a nerve which originates, as one of sensation, from the soft part of the brain, may during its course become condensed in its texture and assume the office of a motor nerve. It is evident, therefore, that he had no idea of any *essential* difference in the character of the various parts of the central organs, or in that of the nervous trunks as derived from them,*

The writings of Galen, as is well known, maintained their sway over medical opinion for nearly fifteen centuries; and men hardly dared to speculate, much less to avow opinions in opposition to those of their great master. With the revival of the spirit of original enquiry, however, new anatomical facts and pathological observations were brought together; and it might have been supposed that physiological inductions would have advanced in no unequal proportion. This was the case in almost every other department but neurology; and it is difficult to assign any other reason for its slow progress than the acknowledged difficulty of the enquiry. One can scarcely help being surprised that, when the functions of lacteal and lymphatic systems, and the double circulation of the blood, were being established, no progress was made towards the elucidation of phenomena so interesting as those connected with the nervous apparatus.

The difference between the motor and sensory functions of the nerves continued to be regarded as a question of speculative interest; and various hypotheses were framed to account for it. No one, however, seemed to think of putting any of these to the test of experiment;

* See Medical Quarterly Review, April, 1834.

indeed, they were generally such as no experiments could have been devised to meet. The names of Vesalius, Fallopius, Van Swieten, and their contemporaries, may be mentioned in this connexion. The attention of physiologists was now more directed, however, to the involuntary motions; and these were regarded by Willis (who was followed on this point by Vieussens, Boerhaave, Du Hamel, and others,) as taking their origin in the cerebellum, whilst the voluntary movements were supposed by them to result from the influence of the cerebrum. In this point an advance towards truth was evidently made; but it was not until some time afterwards that the true nature of the involuntary movements was explained. To Willis we owe much anatomical information regarding the nervous system. The numerical nomenclature of the nerves of the head was established by him; and though this has now had its day, there can be no doubt that it has also had its use. With more precise views in reference to the anatomy of the cerebral nerves, some advance in the knowledge of their uses could scarcely fail of being made; and we accordingly find that from this time the olfactory, optic, and auditory nerves were generally spoken of as nerves of *sensation* only (although it was usually imagined that they bestowed *common* as well as *special* sensibility on the organs to which they were respectively distributed), whilst the third, fourth, and sixth pairs were, from their exclusive connexion with muscles, recognized as specially, if not exclusively, *motor* nerves.* Still no attempt was made to account for the difference in function by more minute researches into their origin, and their connexion with different parts of the central organs.

So vague, indeed, were all the notions entertained upon this subject, that many writers seem altogether to have lost sight of the distinction just alluded to; and the old doctrine which regarded the brain as the

* Willis entered with much more correct minuteness into the functions of the cranial nerves than he has generally received credit for; his numerical nomenclature having been adopted in preference to his physiological. He distinctly states (*Cerebri Anatome*, cap. xxi-xxiii.) that the first and second pairs are nerves of *sensation only;* and that the two next are most especially subservient to motion (*motui potissimum inservire*). The third pair he distinguishes as performing the voluntary movements only of the eye; and he represents the fourth pair as the channel of the involuntary movements, and movements of expression, which he regarded the cerebellum as influencing. He notices the fifth pair as different from the first four, inasmuch as it ministers both to sensation and motion. He regarded all the branches, however, as equally possessed of this double endowment; and believed that the motor influence was subservient not only to volitional but to emotional impulses. He noticed, however, the special destination of the third branch to the masticator muscles; and states his belief that the sense of taste is due to this nerve also. The sixth pair he states to be a muscular nerve simply, and to have for its office the abduction of the eye for the purpose of gaining a backward view, as is done by animals under the influence of fear; whence he regards it as a sort of instinctive nerve. He states that in animals which have a nictating membrane, a twig of this nerve supplies the muscle which draws it across. The portio mollis of the seventh pair he states to be purely a nerve of sense; and the portio dura to be in reality a distinct nerve, although supplying the means by which the *action* of *hearing* may be best performed. He speaks of it as distributed *entirely to the muscles* of the face, and as designed to bring the various organs into co-operative action with that of the auditory sense. With regard to the ninth pair, he states most distinctly (chap. xxix.) that it is the nerve of the motions of articulation, whilst the fifth pair is that of the sense of taste; *and that the reason of this organ being supplied with two nerves is its double function.* As Willis is an author of whom England may justly be proud, it is rather strange that Sir C. Bell has said so little of him.

elaborator of the "animal spirits" which were transmitted by the nerves to every part of the body, still held its place in the schools, as the caustic ridicule of Tristram Shandy abundantly testifies. Even Haller, who contributed so largely to our knowledge of the true influence of the nervous system upon the organism at large, had very confused notions on the subject of the nerves themselves. Thus, although he clearly demonstrates that the first, second, and portio mollis of the seventh nerves were subservient to sensory impressions only, he tells us in another place that he knows not "a nerve which has sensation without also producing motion; the nerve which gives feeling to the finger is that which moves the muscles; and the fifth nerve of the brain branches to the papillæ of the tongue, and also to the muscles." He was perfectly aware that for a *sensation* to be felt, an impression must be propagated along the nerves from the circumference *to the centre;* and that, for a muscular contraction to be produced by a nerve, an impulse must be propagated along the trunk *from the centre* to the circumference; and yet he maintains that the same fibrils may convey either sensory or motor impulses, according to the direction in which they are transmitted.* "The same nerves," he afterwards states, "must evidently preside over both sense and motion; as *we cannot admit a distinction between the two systems of motory and sensitive nerves.* If sense sometimes remains after motion is destroyed, this seems to be because much more strength is required for the latter. Dying people hear and see, when they are incapable of motion." It is justly remarked, therefore, by Sir C. Bell (p. 4), that "Haller, who had traced the opinions of authors with the utmost diligence, gathered nothing from the ancients. The confusion in his mind, as well as in the minds of our most learned physicians and commentators, declared the necessity of having recourse to the volume of nature itself."

Having on a former occasion† given a full account of the opinions of Whytt on the subject of the sympathetic movements, we need not here go over the same ground. Further consideration and acquaintance with Prochaska's writings have even more fully satisfied us of the position we then took—that, in attributing these actions to the "sentient principle," Whytt did not regard *sensation* as necessarily participating in them. He was aware that the spinal cord furnished this sentient principle to the nerves connected with it, and had an action independent of the brain. And he was probably the first to prove, as a general fact, that "the vital and involuntary motions" are dependent upon a *stimulus* propagated to the central organs, and there exciting motor impulses which act through the efferent trunks upon the muscular apparatus. It is quite true that he assigned this as the mode in which some actions are excited (those of the heart and alimentary canal for example), which we now know to be mainly independent of the nervous system; but this error by no means detracts from his merit as having established by solid reasoning that sympathetic actions are not to be explained by "consent of parts or continuity of membranes," but by the interposition of nervous agency operating in the circle just described. Whytt is the first

* First Lines of Physiology, section ccclxxvii—Cullen's edition—Edinburgh, 1786.
† Vol. V., pp. 523-6.

author who is quoted in the "Dates and Documents," and the extract from his work is thus headed,—"Sensation the cause of a conservative motion in the whole or in the separated parts of animals." We must again repeat, that the true import of the term "sentient principle" cannot be understood, unless attention be paid to the phraseology of the time, and especially to that of the Stahlian school, of which Whytt was one of the last supporters. And when he distinctly states, that some stimuli produce vital movements *without consciousness*, we may surely take this as a definition of his meaning. Let it not be forgotten that many physiologists and pathologists, even at the present time, are in the habit of using the term *sensation* itself as synonymous with what is more correctly designated *impression*, and give the term *perception* to the act by which the mind becomes *conscious of sensation.* Now, we would ask, what clearer indication could be given of the intention of a writer to make this use of the term *sensation*, than to state that it is *independent of consciousness?*

The following experiment recorded by Whytt, in his "Observations on Sensibility and Irritability," is a very curious anticipation of those made by Dr. M. Hall. It is made use of by the author as a proof that "the soul" is not confined to the brain, but is present in the spinal cord also. "The late reverend and learned Dr. Hales informed me that, having many years since tied a ligature about the neck of a frog, to prevent any effusion of blood, he cut off its head, and thirty hours after, observed the blood circulating freely in the web of the foot: *the frog also at this time moved the body when stimulated;* but that, on thrusting a needle down the spinal marrow, the animal was strongly convulsed, and immediately after became motionless." Let it not be forgotten, however, what was his view of the operation of the soul on the living system. He regarded it as the universally operating "principle," which governed all its movements and actions; and every other "principle" was a subdivision of it, acting in some peculiar channel. Had the term "reflex function" been proposed to him for that portion of it which operated through the spinal cord, we are persuaded that he would have adopted it on account of its appropriateness, and set it down as an action of "the soul," just as many physiologists of the present day would do in regard to the "vital principle." The state of anatomical knowledge at that time did not enable him to limit the participation of the nervous centres in the actions of this class to the spinal cord and medulla oblongata, as we can now do; but no one who dispassionately reads his writings with due allowance for his phraseology, can fail to allow that, in all essential points, we have given a fair representation of his opinions; and these, although comparatively little known elsewhere, have been annually made the subject of prelection in the Edinburgh School, from the days of Cullen to the present time.

During the latter part of the eighteenth century, many important contributions were made to the anatomy and physiology of the nervous system. The discovery of the general fact that the ganglia of the spinal nerves are formed on their posterior roots only, and that the fibres derived from the anterior roots pass over them, seems to have been made, or at least first enunciated by Dr. Monro, *primus*, in his valuable work on the nerves. As to the functions of these ganglia, great difference of opinion prevailed.

By Dr. Johnstone it was conceived that they were interposed for the purpose of cutting off sensation in those operations of the nervous system which produce the involuntary movements. A remark of Dr. Monro's on this hypothesis shows his complete ignorance of the respective functions of the two roots. "That ganglia do not serve," he says, "to render motions independent of our will, as an ingenious author has supposed, is evident, without observing more than that all the branches of the fifth pair, and the posterior half of all the spinal nerves of the voluntary muscles, pass through ganglia." On this Sir C. Bell justly remarks—"If I had ascertained nothing more than that no motor nerve passes through a ganglion, the observation would have been important towards the true doctrine of the nerves."

Dr. Monro's mistake regarding the fifth pair was corrected by other anatomists. Santorini and Wrisberg had observed its two roots; Prochaska and Soemmering noticed that the anterior root passes by the ganglion, and enters the third division of the nerve; and both were struck by its conformity in this respect with the spinal nerves. Paletta went further; for, tracing the third branch principally to the muscles of the jaw, he conceived that the anterior root, which enters it alone, is a purely muscular nerve; "and when," says Sir C. Bell, "we should have expected that he was about to discover the truth, he acknowledges that he does not know what to make of the other branches of the fifth nerve." It appears to have been the general opinion that a plurality of nerves in any organ was simply to provide it a good supply of nervous agency, so that if one failed another might supply its place. Soemmering seems to have partaken of this idea; yet he points out that one nerve might give motion to the tongue, and another sensation: whence a man might lose his taste and yet move his tongue as before.* Scarpa dwelt with great minuteness on the anatomy of the roots of the spinal nerves, and had an idea that there might be a functional difference between them. But his idea was wrong. He asks, "Is the posterior root a proper and peculiar kind of nerve, belonging exclusively to the spinal marrow, whilst the anterior root is a cerebral nerve?" So far as this speculation was regarded, then, it might truly be said that this illustrious anatomist and surgeon "left the system doubly confounded."

We see, then, that those who examined the anatomical relations of the nerves with the greatest minuteness were ignorant of the physiological bearing of the facts they revealed; or that, at most, they derived from them one or two inferences which had only a very limited application. On the other hand, those who held the notion that the sensory and motor nerves are distinct, were content with the mere speculation, and did not attempt to support it either by the evidence of anatomical relation, or by that with which experiment might have supplied them. Pouteau, whose posthumous works were published in 1783, revived, on pathological grounds, this ancient opinion; he remarked, however, that it had been long abandoned by anatomists. He supposed that the nerves of sensation come from the cerebrum (or rather proceed to it), and those of volition from the cerebellum—an opinion which is not without its advo-

* This statement, like that of Willis, Sir C. Bell seems to have overlooked in his generally candid, though deficient, historical summary. It will be found, in the *Lehre von Hirne und von den Nerven*, p. 255.

cates at the present time. But he went no further than this vague surmise. Dr. Darwin, in his remarkable Zoonomia, points out that a morbid sensibility to warmth is occasionally observed in paralysis, although the sense of touch be not morbidly acute, or be actually impaired; and he thence infers, not only that there are nerves of sensation distinct from those of motion, but nerves for the sensation of temperature distinct from those of common sensation. But here his speculations ended.

During the period we have been just considering a very important change took place in the views of anatomists, in regard to the sympathetic, ganglionic, or more truly the *visceral* system of nerves. This had been formerly described as a kind of off-set from the cerebro-spinal, descending from its origin in the fifth and sixth cerebral nerves, and deriving reinforcements from the spinal nerves. Previously, however, to the time of Bichat, juster views of its nature were prevalent; and its title to the character of a distinct system was generally recognized. Availing himself of this, and of a certain superficial analogy between the chain of ganglia it presents and that which constitutes the central apparatus in the articulated classes, he propounded his specious view of its functions as the nervous system of organic life. This, to a certain extent, is correct; since we are fully assured that its operation is confined to the organs concerned in the purely vital functions. But we cannot agree with Bichat that all the changes to which these organs are subservient are regulated by it, as he supposed, whilst the cerebro-spinal system has no participation in them. And further, the untenable nature of his theory is evident from the very analogy he adduced in its support, since he attributes to what he regards as the analogue of the ganglionic system in the articulated classes, the functions of sensation and voluntary motion which they possess in a higher degree than any other invertebrata. With all his errors, however, Bichat did much to fix attention on the important fact that there is a plurality of function and a corresponding plurality of structure, in the nervous system.

We must not omit to point out, also, the advance made by Prochaska towards the truth, in relation to the excito-motor system of nerves. Our readers will remember that his ideas on this subject were only brought to our notice after our former historical summary had been printed. At the conclusion of the same number (Vol. V., p. 623,) we gave from the original the whole chapter relating to it; and we shall take this opportunity of making a few observations on its import. In the first place we shall repeat the remark we there made, that Prochaska does not "notice the subject merely incidentally or as a matter of no importance, but that he clearly and explicitly announces the phenomena as depending on a distinct and important property of the nervous system, to which he devotes a whole chapter of his treatise." We desire that it should be borne in mind that the "complete anticipation" of which we there spoke related to the doctrine of the "reflex function of the spinal cord," which was the whole extent of what we were then inclined to admit as true in Dr. M. Hall's views; and we think it will appear that almost all that is deficient in the views of Prochaska on this subject is supplied by the discovery of Sir C. Bell. Now we by no means insinuate that Dr. M. Hall was acquainted with Prochaska's doctrines, when he commenced his researches. In fact, we believe his to be one of the cases in which a little more knowledge of what had been previously taught would have greatly assisted him, without depriving him of his claim as a discoverer. But having the work containing them at his command, and professing in his second memoir to give a full account of the doctrines of other physiologists, we are surprised at the omission of all reference to them. We must not be understood as asserting that the *general* state of knowledge on the subject was much affected by Prochaska's publication. In fact, it seems to have excited little attention at the time, and to have been subsequently altogether lost sight of.

Now in his case, as in that of Whytt, we must adopt *his own* explanation of his terms, or we shall be altogether misled. The title of this chapter is an enquiry into the seat and functions of the *sensorium commune.* In modern writings this term is applied to the part of the nervous system where *impressions* become *sensations;* and if we set out with this notion we should find it difficult to interpret any part of his doctrines; but he takes care to define his meaning in the very first paragraph, where he explains the *sensorium commune* to be that part of the nervous centres at which *external impressions*, conducted to it by different nerves, give rise to *certain and determinate motions* by *respondent motor nerves.** To avoid confusion, we might substitute the term *centre of reflexion* for sensorium commune; our author having distinctly stated that he applies the latter term in accordance with received notions, to the *locus* in which *impressions are reflected* through the motor nerves.

This centre of reflexion he expressly limits to the *spinal cord, medulla oblongata, crura cerebri*, and *cerebelli*, and *part of the thalami optici.* The cerebrum and cerebellum he states to be the instruments which the mind immediately uses for its peculiar actions. He adverts to experiments on decapitated animals as proving the independent power of the spinal cord, which he speaks of as the *seat of the consent between the sensory*† *and motor nerves.*

Prochaska then goes on to point out that this *reflexion of sensory into motor impressions* does not follow mere physical laws, but *peculiar laws written as it were by nature on the medullary pulp of the sensorium;* and these laws he shows to be the preservation of the body from external injury, by the production of motions in respondence to external impressions, *tending to ward off and remove the source of injury;* and also the conservation of the body by the reflection of external impressions into motions tending to its benefit. Three examples are given, than the first two of which none could be more applicable; the motion of the respiratory muscles in sneezing, produced by irritation of the schneiderian membrane and directed to remove the source of irritation; the cough produced by irritation of the wind-pipe by a crumb of bread or a drop of liquid;—and the *involuntary* closure of the eyelid when the finger is brought *near* the eye‡, which is rather of the *emotional* character.

* All the phrases in italics are literal translations of Prochaska's expressions.

† That we are not to understand this term as involving *sensation* will be obvious from what follows. It is manifestly equivalent to *afferent.*

‡ One of Sir C. Bell's cases is a very interesting illustration of the double provision made for the protection of this important organ. The patient had amaurosis of one

He next states that, as the principal function of the sensorium commune consists in the reflexion of sensory into motor impressions, it is to be remarked *that this reflexion may take place whether the mind be conscious or unconscious of it.* Now it is quite true that, like Whytt, he seems to have attributed the movements of the heart and alimentary canal to reflexed action; and he has used these as illustrations of the above position. But he does not speak certainly on this point, and puts it as a query whether these actions are not the result of impressions reflexed through the visceral ganglia. This is a curious anticipation of Mr. Grainger's doctrine formerly referred to; but he does not rest his assertion upon these, for he refers in proof of it to the motions of apoplectic patients, *in whom all consciousness is suspended;* also to the convulsive movements of epileptic patients, *in which the sensorium commune acts without consciousness of the mind;* and to those which are witnessed in profound sleep, as well as to those of decapitated animals.* "All these actions," he continues, "arise from the organization and physical laws proper to the sensorium commune, and are *spontaneous and automatic.* Those of which the mind is conscious are either such as it cannot control by an effort of the will, or such as it can restrain or prevent at pleasure. The former, as they are ruled by the sensorium commune alone, in as far as it does not depend on the mind, are not less to be regarded as *automatic* actions than those which are performed unconsciously; such are sneezing from a stimulus applied to the nostrils, cough from a stimulus applied to the trachea, vomiting from irritation of the fauces, or from an emetic, tremor and convulsions in chorea S. Viti, and in the paroxysms of intermittent fever, &c. But the actions which the mind directs and moderates by its power, although the sensorium commune has its part in producing them, we call, nevertheless, animal, not automatic."

It is perfectly evident, then, that Prochaska had a very distinct idea of the *function of the spinal cord and medulla oblongata as a centre of reflected actions;* and that he specified the principal classes of these, referring them all to the excitement of impressions. We do not see what essential difference there is between this doctrine and that of the reflex function of the spinal cord as *first* propounded by Dr. M. Hall. The *subsequent* idea of a system of spinal or excito-motor nerves, distinct from the cerebral or sensori-volitional, is unquestionably due, we again repeat, to the latter gentleman alone. Indeed, it may be said to be more original with him than the idea of the distinctness of the sensory eye, and anæsthesia of the other. When the finger was brought *near* the blind eye, no effect was produced on the muscles; but when the surface of the eye was *touched,* a reflex action through the afferent portion of the fifth pair and the portio dura excited the closure of the lid by the orbicularis. In the other eye, winking was occasioned by the *approach* of the finger; but, if the lid were held open until the surface of eye was *touched,* no contraction of the orbicularis was excited—the ophthalmic branch of the fifth being paralysed. The first, however, is rather an emotional action, intermediate between the voluntary and the simply reflex. The second is unquestionably reflex only.

* We do not see that Dr. M. Hall brought any further *proof* of this position, until he was able to adduce cases of paralysis in the human subject, in which reflex actions could be excited without sensation. Sir C. Bell had previously shown that the respiratory movements could be excited in muscles which were powerless as instruments of the will.

motor and motor nerves was to Sir C. Bell, since the latter had been frequently brought forward as a speculation; and as we have just seen, was distinctly stated (as a speculation only, however,) by Prochaska.

We must not forget to state that this chapter from Prochaska is quoted in full in the "Documents and Dates" and is accompanied by a translation which is generally faithful, though occasionally deficient in clearness, seemingly from the desire to keep as close as possible to the words of the original.

The doctrine of plurality of functions in the nervous system was carried to a much greater extent by Gall. Whatever may be the opinions of anatomists and physiologists respecting phrenology, it cannot be questioned that we owe much to Gall, and to those who worked under his guidance, for the rapid advance which was made about this time in the anatomy of the nervous centres, and in the knowledge of their true relations to each other. Gall had a strong conviction of the distinctness of the motor and sensory nerves; and used this as an illustration of his peculiar doctrines In the first volume of his Anatomy of the Nervous System, published in 1810, he urged this upon his adversaries; and refers to the fact of the fifth pair, which confers both sensibility and motive power, having three distinct roots, as an argument in favour of his doctrine of *plurality*. Perhaps the most important contribution which he made to our physiological knowledge was his demonstration of the true nature of the cerebral ganglia. By his predecessors, the brain had been regarded as the great centre of nervous energy; and the spinal cord as a sort of appendage or prolongation of it, possessed of some amount of independent power. By his researches in Comparative Anatomy, Gall was led to the belief that the spinal cord is really the central organ, and that the cerebellum and cerebral ganglia are developed upon it in proportion to the required predominance of their respective functions. He succeeded to a great extent in unravelling the structure of the latter; proving that they consist in part of fibres connecting the cortical substance with the medulla oblongata, and partly of commissural or connecting fibres proceeding in all directions, and uniting the different portions of the cortical substance to others in the same and opposite hemisphere. At the same time Reil was occupied with similar enquiries, specially directed towards the structure and functions of the cerebellum, and these, according to Dr. Elliotson, (*Physiology*, p. 330,) were suggested by Gall, and prosecuted on his plan.

We now approach the period when Sir C. Bell commenced his researches. There can be no doubt that his views were formed in ignorance of a great part of what we have thus brought together, and especially of the accounts which had been given of the anatomy of the roots of the fifth pair, and the analogy which had been pointed out between this and the spinal nerves. It must be remembered that, at the period of which we speak, there was much less communication among men of science than at present. A discovery of any kind was a long time in finding its way from one country to another; and the small facility given by the periodical press of those times to the diffusion of knowledge tended still more to restrict it within narrow limits. That Sir C. Bell was unacquainted with the statements and opinions of Paletta, Soemmering, and Prochaska, no one can have any reasonable doubt; since the knowledge of them would not only have materially aided his enquiries, but would have saved him from the only serious error in which he has not corrected himself. That these statements had not excited general attention is sufficiently evident from the fact that Meckel took no notice of them in an elaborate memoir which he composed on this nerve; and that even he omitted to mention its origin by a double root. John Hunter, too, made the same omission, in a description which he had occasion to give of it. "In short," says Mr. Shaw, "I may observe, that I have not succeeded, after a careful search, in finding any single work in the English language, in which allusion is made to the true structure of the origin of this nerve, before Sir C. Bell commenced his investigations." That he should know nothing of Gall's Anatomy is not surprising when we recollect the distrust with which phrenology was at first received.

But we must not yet enter upon the history of Sir C. Bell's discoveries. A claimant remains to be disposed of, whom many of our readers perhaps never heard of but as the author of "Intermarriage," "Woman Physiologically Considered," "Theory of Beauty," and other works of similar erotico-physiological character. His title to discovery is thus set forth in the "Dates and Documents:"

"To Mr. Walker's papers, then, we first proceed; as, whether they be right or wrong as to function, direction, or course, they are certainly those of by far the earliest writer who speaks of *nerves, spinal columns, and cerebral masses of sensation,—of the continuation of the action thence originating through the cerebrum,—and of other cerebral masses, spinal columns, and nerves of volition,—thus forming the great circle of nervous action and influence.*—This appears to be at least the first part of the discovery, 'the conception or idea,' as described by Professor Whewell." (p. 13.)

The first part of Mr. Walker's claims is that of assigning a function to the cerebellum, which it seems he did in a "Table of a Natural System of Medical Science," published in 1808. The cerebrum is here set down as the organ of perception, and the cerebellum as the organ of volition. This dogma, enunciated as it is without the support of a particle of evidence, is nothing more than a revival of the doctrine of Pouteau. We do not mean that Mr. Walker must necessarily have been acquainted with this; or that, if he had proved it by additional facts, he would not have been entitled to the merit of the discovery; but in its insulated form it is a mere speculation, and must rank as such.

We are next presented, however, with a series of extracts, from a paper published by Mr. Walker in April, 1809; and upon some parts of these we must bestow a little more attention. The first passage we shall quote will serve as a specimen of the philosophy of its author. "*That sensation* belongs entirely to the organs of sense, is *clearly proven by this, that several animals possessing a brain retain sensation* (*in this case improperly termed irritability*) *unimpaired long after it is removed.*" (p. 20.) We are afterwards informed that "*all the other intellectual functions are modifications of sensation.*" Again, "as sensation exists without volition, and as almost all nerves arise by distinct filaments, I am of opinion that, wherever a part, having both sensation and motion, is supplied from one nervous trunk, that trunk envelopes both a nerve of sensation and one of motion." Many had anticipated Mr. Walker in the enunciation of that opinion; and as long as it remained unsupported by evidence, no advance was made. In a little work now before us, entitled Anatomia Britannica, and published in 1808, we find the following passage, which, succeeding as it does an account of the two principal terminations of the nerves—in muscles, and in sensory organs,—is to our minds quite satisfactory as to the opinion of its author. "Though the fibres in a nervous cord are firmly connected, and frequently nerves join into one trunk, or into the same ganglion, yet the sensation of each part of the body is so very distinct, and we have so much the power of moving the muscles separately, that, if the nerves are principal agents in these two functions, we have reason to believe that there is no union, confusion, or immediate communication of the proper nervous fibrils, but that each fibre remains distinct from its origin to its termination."* We have quoted this passage with a view of also showing that the statement made by Sir C. Bell in his first pamphlet,—as to the prevailing doctrine of the anatomical schools being that *the same* nerves which convey sensory impressions *to* the brain transmit motor impulses *from* it,—is rather too sweeping a generalization. But this is a matter of comparatively little importance; since neither one doctrine nor the other was ever *proved.*

We return, however, to Mr. Walker. His next assertion is that the *cerebellum and medulla oblongata* constitute the *organ of volition.* This opinion he states to be supported by "numerous observations;" but we find none which bears closely upon it, and no reference to experiment. Of the novelty of the dogma regarding the cerebellum we have already spoken; and the absurdity of limiting the function of the spinal cord to volition is self-evident. We are afterwards told that "the only apparent difference between the nerves of sensation and those of volition is, that their motions take place in different directions. The latter, therefore, may be said to resemble the arteries; the former the veins." The first part of this passage is a mere hypothesis; the second, which is printed in capitals on account of its supposed importance in establishing Mr. Walker's claims to discovery, contains nothing new. Hartley had long anticipated him in the speculation. In his Essay on Man may be found a beautiful hypothesis (of which this is the basis), plausibly enunciated, and pursued into extensive application. This we should recommend to Mr. W.'s perusal, as it may furnish him with some valuable hints for his next work.

A second paper by Mr. Walker next presents itself among the Dates and Documents, of more pretensions than the former one. The title of this is a little remarkable—"New Anatomy and Physiology of the Brain in particular, and of the Nervous System in general." It is contained in the Archives of Universal Science; and the date of its publication is July 1809. This cannot be passed over as summarily as the other; for it certainly does contain a very remarkable *anticipation* of Sir C. Bell's views;—an anticipation, however, which was dogmatic only, which was never subjected to the test of experiment, which never led the author fur-

* Vol. iii., p. 238. Since this paper has been in type we have learnt that Mr. Walker is himself the author of the Anatomia Britannica. He was, however, by no means singular in the opinion there enunciated.

ther, and with which we have no proof that Sir C. Bell had become acquainted, when he commenced his more successful researches. It is due to the author, however, to present it in full.

"The medullary matter may be traced as continued from the portions of many nerves which join the two anterior columns of the spinal marrow, upward through these columns to the inferior fasciculi of the medulla oblongata, forward through the crura cerebri, outward and upward through the corpora striata, and the hemispheres of the cerebrum. From the hemispheres it passes posteriorly, backward, inward, and downward through the thalami, backward through the striæ inferior to the nates and testes, and backward and upward through the processus cerebelli ad testes, or the anterior peduncles of the cerebellum to the substance of the cerebellum itself. Lastly, from the cerebellum it passes downwards by the corpora restiformia, superior fasciculi of the medulla, processus cerebelli ad medullam, or posterior peduncles of the cerebellum to the posterior columns of the spinal marrow, and the remaining portions of the numerous nerves which join it. Thus the medullary fibres form a most remarkable circle, of which this is the direct course." (pp. 32-3.)

In this description, which is by no means anatomically correct, Mr. Walker was anticipated by Gall, who had been for some years in the habit of demonstrating the course of the fibres of the brain in a manner generally similar. Let it be observed that he had nothing but speculation to guide him in assigning to the two sets of fibres an *ascending* or *descending* course; and that his speculation was wrong. It is in his physiological explanation that we meet with the chief novelty, that of assigning motor and sensory functions respectively to the two origins of the spinal nerves; and here, too, we find the basis upon which the assignment was founded.

"But it may be questioned by which nerves, columns, and cerebral masses, the action ascends to the brain, and by which it descends to the muscles. Fortunately, here nature also directs us. Several nerves of mere sensation join the anterior masses; hence they must be the ascending: one nerve of mere locomotion proceeds from the posterior masses; hence they must be descending—for sensation, as already said, must ascend to, and volition must descend from, the sensorium commune.

"Thus, then, it is proved to us, that medullary action commences in the organs of sense; passes, in a general manner, to the spinal marrow, by the anterior fasciculi of the spinal nerves, which are, therefore, nerves of sensation, and the connexions of which with the spinal marrow or brain must be termed their spinal or cerebral terminations; ascends through the anterior columns of the spinal marrow, which are, therefore, its ascending columns; passes forward through the inferior fasciculi of the medulla oblongata, and then through the crura cerebri; extends forward, outward, and upward through the corpora striata, and reaches the hemispheres of the cerebrum itself. This precisely is the course of its ascent to the sensorium commune. From the posterior part of the medulla of the hemispheres, it returns by the thalami, passing backward, inward, and downward; flows backward in the fasciculi under the nates and testes; backward and upward through the processus cerebelli ad testes, or anterior peduncles of the cerebellum; and thus reaches the medulla of the cerebellum itself. From the cerebellum, it descends through the posterior column of the spinal marrow, which are, therefore, descending columns; and expands through the posterior fasciculi of all the nerves, which are, therefore, the nerves of volition, and the connexions of which with the spinal marrow or brain must be termed their spinal or cerebellic origins." (pp. 35-6.)

Now it cannot for a moment be questioned by any one who reads this passage, that its author had the *idea* of a difference in the functions of the fibrils of the same nervous trunk, depending on a difference in the part of the central organs with which they are connected; and that this difference is manifested in the spinal nerves by the connexion of each at its root with two distinct columns of the spinal cord, one a prolongation of the cerebrum, and the other of the cerebellum; whilst in the head there existed nervous trunks separately appropriated to these functions, and directly connected with the two central organs. But the *value* of the idea depends upon the evidence adduced in its support, and the use subsequently made of it. Let us enquire what amount it can claim on these grounds. Mr. Walker's idea of a distinctness in the functions of the roots of the spinal nerves is founded upon two suppositions. First, that the anterior and posterior columns are continuous with the cerebrum and cerebellum respectively. This, every anatomist now knows to be incorrect; both columns being connected with each organ. Secondly, that the cerebrum is the organ of sensation, and the cerebellum of volition. This opinion has now been abandoned by every physiologist of reputation; the few who maintain that such an antagonism exists between the functions of the two masses, being of opinion that the cerebellum is the organ of sensation, and the cerebrum of volition; and the greatest number adopting the opinion of Rolando and Flourens, that the cerebral hemispheres are the organs both of sensation and volition, whilst the cerebellum is the organ by which the separate motive impulses (whether voluntary or involuntary) are harmonized and controlled, so as to produce the more complex actions. Whatever be the shades of difference on the latter point, there is little or none on the former. If *either* of the two positions on which Mr. W's. hypothesis was founded, be proved to be untenable, the doctrine falls to the ground, since one support is nothing without the other; but when *both* are shown to be fallacious, not a shadow of a proof upholds it.

Still the *idea* may be regarded as an ingenious speculation; and had it been subjected to the test of experiment, it might have conducted its author to the truth. But has it been thus fruitful? So far from it, that the author, relying upon its self-evident merits, took no pains to perfect and substantiate his supposed discovery; and nothing more of him or it (as far as we know) was heard by the scientific world in this country, until the publication of his work on the subject in 1834, in which he accuses Sir C. Bell, and all who have been engaged in the same enquiry, of having stolen his discovery from him.

We have shown then, that his *idea*, however ingenious as a speculation, had no real value either in itself, or as having led to other discoveries. We will suppose for a moment, that Bell, Magendie, Mayo, &c., had never existed, and that Mr. Walker's opinions had gained the public credence to which their author considers them entitled: unless some more acute physiologist had corrected the error, we should have been now in a worse position than when everything was regarded as uncertain; and we should have had more difficulty in finally attaining truth, since every one knows that the overthrow of old doctrines is often a far more laborious process than the establishment of new ones. The *only* way in which it seems to us possible that Mr. Walker's speculations can have contributed to the advancement of science, is that they may have suggested to Sir C. Bell the course of investigation which he has followed up with such gratifying success. But, independently of other considerations we feel assured by the internal evidence of Sir Charles's first pamphlet, that his views were entirely original.

We now enter upon the period embraced by Mr. Shaw's history, of which we shall in future make frequent use, commenting freely both upon its statements and its deficiencies. The first point we shall advert to, is one which has already come under our review, and which we shall now merely notice. It is the statement of the author, coinciding with that of Sir C. Bell, as to the degree of previous ignorance on the subject. We have shown that many physiologists entertained at different times the opinion that the motor and sensory fibrils were distinct, and terminated in different parts of the nervous centres; and that this was expressed in an anatomical work published at that very period. We do not think that it derogates in the least degree from Sir C. Bell's merits as a discoverer to make this admission; and we think it better, that it should be stated broadly and fully, in order that claims to more complete anticipations may be at once refuted. It is only by gaining clear notions on the state of previous knowledge that the true merits of any discoverer can be appreciated. That the floating opinions to which we have just referred could not be regarded as *knowledge*, is evident, from the great diversity which existed amongst them. One speculation, however, had evidently more influence on Sir C. Bell's mind than he was at first aware of; it is that of John Hunter, that the nerves have different functions, and that the complexity of their distribution will be explained when these are thoroughly known. Sir Charles has duly acknowledged his obligation to his great master, in a note to the second paper on the nerves of the orbit. (3d edition, p. 188.)

We entirely concur with Mr. Shaw in the statement which he has very properly prefixed to his historical details, that Sir C. Bell's chief merit as a discoverer consisted in directing attention to the *roots of the nerves*, as the part of the whole nervous system most adapted to afford *information to the experimenter.* If it be true that these have different functions, the physiologist is enabled to ascertain them by rigid tests; and he may thus more clearly determine the functions of the mixed trunk into which they unite, and by tracing further back, infer the office of the portion of the nervous centres with which they are respectively connected.

"Here then is a simple explanation of the principle on which all these new discoveries have been based. It consists, I repeat, in supposing that, to investigate the functions of the nervous system successfully, we must devote our attention, not to the trunks, as was formerly done, but to the roots of the nerves. Accordingly, whoever was the first to suggest and follow out that new method of prosecuting the subject, must be declared the true originator of the recent improvements in this department of physiology. It is by the test of who did the most to establish this law? that we must decide to whom we are indebted for these discoveries." (*Narrative*, p. 7.)

Now here we feel that Sir C. Bell can fairly meet Mr. Walker. Did the latter enunciate this principle? It may be said that it flows self-evidently from his doctrine. But we know that the most self-evident inferences often remain undeduced. Was this the case? We answer without hesitation that it was. During *twenty-five years'* investigation of the subject, Mr. Walker made but *one experiment,* the results of which coincided with those obtained by Sir C. Bell; and finding that this method revealed facts inconveniently opposed to his opinions, Mr. W. very judiciously abandoned it.*

Some confusion exists as to the time when Sir C. Bell first propounded his new views. In his own volume, as well as in Mr. Shaw's history, this date of the *printing* and *circulation* of the "Idea of a New Anatomy of the Brain" is fixed at 1811; whilst in Mr. J. Shaw's Essay on Paralysis of the Nerves of the Face,† it is stated as 1809; and this statement was repeated in the discussion with Magendie. This requires some explanation; but it is evident that, if there be any real uncertainty arising from the want of a date attached to the paper (which was never published), Sir C. Bell and his present historian have erred on the safe side. We have long had a curiosity to meet with this essay. The first ideas on which a grand discovery is founded are always interesting; and to trace the progress of their development is a most instructive exercise. Who that has read the account of the investigations which conducted Davy to the invention of the safety-lamp, has not felt a sympathetic delight in the gradual unfolding beneath his search of the important principle which is there so beautifully applied? We must confess that we should have been glad to see this essay prefixed to the collection of memoirs, read before the Royal Society. The very fact of its not having been published adds to the reasons why it should have appeared there; since, if it be appealed to for historical data wherewith to establish Sir C. Bell's claim to public reputation, the public have a right to know *all* that it contains. We still more regret that Mr. A. Shaw has not thought fit to publish it entire, but has contented himself with such extracts as suited his purpose. These extracts, we feel it our duty to state, unequivocally, *do not give a fair view* of the essay. When we rose from the perusal of this part of Mr. Shaw's history, it was with the feeling of unmingled astonishment that Sir C. Bell's original opinions could have been so boldly misrepresented, as it would seem from it that they have been. But when an opportunity was furnished us by the "Dates and Documents" of making ourselves acquainted with the whole paper, we at once perceived the sources of those statements, and saw with regret, as far as Mr. Shaw is concerned, and with satisfaction in regard to the individual whom he chiefly criminates, that they could scarcely be regarded as misrepresentations, although they erred in taking a one-sided view of Sir C. Bell's opinions. As to Sir C. Bell himself, we gladly state that the perusal of this paper, with all its errors and imperfections, has *not in the least degree* lowered our estimate of his merits as a discoverer,—and for this reason: the *principle* is there plainly and unequivocally enunciated which guided all his subsequent researches. They may fairly, therefore, date back to this period; and we also trace that philosophic spirit, so opposed to the dogmatism we have elsewhere encountered, which prevented him from hasty decision, whilst it also pointed out the road to more certain information. We should not do

* We make this statement on the authority of Dr. Fletcher, (*Rudiments of Physiology,* Part ii., *b*, p. 102,) whose statements, being referred to by the editor of the "Dates and Documents," may, we presume, be regarded as authentic.

† Medico-Chirurgical Transactions, vol. xii., p. 149.

him justice, if we did not quote a passage or two in illustration of this general character. The first is from his introductory remarks.

"When in contemplating the structure of the eye, we say, how admirably it is adapted to the laws of light, we use language which implies a partial, and consequently an erroneous view; and the philosopher takes not a more enlarged survey of nature when he declares how curiously the laws of light are adapted to the constitution of the eye. Thus, creation of which we are a part, has not been formed in parts. The organ of vision, and the matter or influence carried to the organ, and the qualities of bodies with which we are acquainted through it, are parts of a system, great, beyond our imperfect comprehension, formed as it should seem at once in wisdom; not pieced together like the work of human ingenuity. When this whole was created (of which the remote planetary system as well as our bodies, and the objects familiar to our observation, are but parts,) the mind was placed in a body not merely suited to its residence, but in circumstances, to be moved by the materials around it; and the capacities of the mind, and the powers of the organs, which are as a medium betwixt the mind and the external world, have an original constitution framed in relation to the qualities of things." (*Documents and Dates*, p. 41.)

These remarks are followed by some sagacious distinctions between the different kinds of sensation; the object of which is to show that these depend, not only on the difference of the organ in which the impressions originate, but on the different endowments of the nerves conveying them, and on the diversity of function in the portions of the central organ with which they are respectively connected. These observations appear intended to introduce and illustrate the main argument of the essay, that the brain, instead of consisting of parts having similar endowments, is composed of numerous organs, ministering to distinct offices; and they conclude with the following admirably-expressed and philosophical views:

"Thus we see that, in as far as is necessary to the great system, the operations of the brain, nerves, and muscles, are perfect from the beginning; and we are naturally moved to ask, might not the operations of the mind have been thus perfect and spontaneous from the beginning, as well as slowly excited to action by outward impressions? Then man would have been an insulated being, not only cut off from the inanimate world around him, but from his fellows; he would have been an individual, not a part of a whole. That he may have a motive and a spring to action, and suffer pain and pleasure, and become an intelligent being, answerable for his actions, sensation is made to result from external impression, and reason and passion to come from the experience of good and evil; first, as they are in reference to his corporeal frame, and finally as they belong to the intellectual privations and enjoyments." (*Ib.*, p. 47.)

The study of men's characters from their writings is a most interesting employment. When pursued with acuteness and discrimination, it often leads to far juster impressions than personal intercourse produces; such is our feeling in the present instance. What mind capable of appreciating such a passage as that just quoted would not say—"Set that man upon a right track, and he will be the discoverer of important truths?" Let us see whether its author succeeded in finding such a path.

The whole object of the essay is evidently the establishment of the doctrine of a plurality of endowments in different nervous trunks, and in different parts of the same trunk; these endowments being possessed by them in virtue of their connexion with different portions of the central organs. In this manner it was conceived by the author that the complexity of the distribution of the nerves, their union with one another, and many similar points of interest and difficulty to the anatomist, might be satisfactorily explained; but as long as he confined himself to mere argument and speculation, it is manifest that he only advanced upon the doctrines of others in the additional reasons he adduced. This, however, was not all. Bell perceived the deficiency, and his sagacity pointed out the means of remedying it. Our next extract gives his own statement in full, and it is sufficiently apparent, that his mode of investigation was completely opposed to the dogmatic assumption of Mr. Walker, who reasoned downwards to the roots of the nerves from the false position with which he started. It will be observed that, in common with Mr. W., Sir C. Bell entertained an incorrect, or rather an imperfect, view of the connexion of the brain and spinal cord; and this, as we shall hereafter see, was one cause of the errors of detail into which he was at first led.

"In thinking of this subject, it is natural to expect that we should be able to put the matter to proof by experiment. But how is this to be accomplished, since any experiment direct upon the brain itself must be difficult, if not impossible? I took this view of the subject. The medulla spinalis has a central division, and also a distinction into anterior and posterior fasciculi. corresponding with the anterior and posterior portions of the brain. Further, we can trace down the crura of the cerebrum into the anterior fasciculus of the spinal marrow; and the crura of the cerebellum into the posterior fasciculus. I thought that here I might have an opportunity of touching the cerebellum, as it were, through the posterior portion of the spinal marrow, and the cerebrum by the anterior portion. To this end I made experiments, which, though they were not conclusive, encouraged me in the view I had taken. I found that injury done to the anterior portion of the spinal marrow, convulsed the animal more certainly than injury done to the posterior portion; but I found it difficult to make the experiment without injuring both portions.

"*Next, considering that the spinal nerves have a double root, and being of opinion that the properties of the nerves are derived from their connexions with the parts of the brain, I thought I had an opportunity of putting my opinion to the test of experiment, and of proving at the same time that nerves of different endowments were in the same cord, and held together by the same sheath.*" (*Ib.*, pp. 50-1.)

This is the grand principle of Bell. It seems simple enough when it is pointed out to us; but had any one even hinted at it before? Such importance do we attach to it, that we consider its author as entitled to the merit of whatever discoveries might be made by following it out, excepting, of course, that share which belongs to the ingenious and discriminating experimenter. Supposing that Sir C. Bell had died or had been prevented from pursuing his physiological researches, before he had himself established any detailed results, would it not have been fairly asserted that he was *de facto* the author of whatever improvements might be introduced into the science by the adoption of his method? To us it seems as if he had thus unlocked a secret spring which allowed the chest to be opened and its contents examined, or found the concealed thread by which the labyrinth of truth might be explored. But let us again refer to his essay.

"The spinal nerves being double, and having their roots in the spinal marrow, of which a portion comes from the cerebrum and a portion from the cerebellum, they convey the attributes of both grand divisions of the brain to every part, and therefore the distribution of such nerves is simple, one nerve supplying its distinct part. But the nerves which come directly from the brain, come from parts of the brain which vary in operation; and in order to bestow different qualities on the parts to which the nerves are distributed, two or more nerves must be united in their course or at their final destination; hence it is that the first nerve must have branches of the fifth united to it; hence the portio dura of the seventh pervades everywhere the bones of the cranium to unite with the extended branches of the fifth; hence the union of the third and fifth in the orbit; hence the ninth and fifth are both sent to the tongue: hence it is, in short, that no part is sufficiently supplied by one single nerve unless that nerve be a nerve of the spinal marrow, and have a double root, a connexion (however remotely) with both the cerebrum and cerebellum. Such nerves as are single in their origin from the spinal marrow will be found either to unite in their course with some other nerve, or to be such as are acknowledged to be peculiar in their operation. . . . . .

"Understanding the origin of the nerves in the brain to be the source of their powers, we look upon the connexions formed betwixt different nerves, and the combination of nerves in their passage, with some interest; but without this, the whole is an unmeaning tissue. Seeing the seeming irregularity in one subject, we say it is accident, but finding that the connexions never vary, we say only that it is strange, until we come to understand the necessity of nerves being combined, in order to bestow distinct qualities on the parts to which they are sent." (*Ib.*, pp. 51-3.)

What can be conceived more clear and explicit than this statement of the *general principles* on which Sir C. Bell desired that subsequent enquiries should be pursued? Had he been desirous of preventing any others from entering upon it, he would have scarcely placed his "Idea" in the hands of those members of the profession in Great Britain and Ireland, whom he thought most likely to aid in its development. We can scarcely help feeling surprised at the apathy with which it seems to have been received; there being no evidence that any one partook in the subsequent enquiry but Sir C. Bell's own pupils. We shall presently show that he completed the *general structure* for which he here laid so good a foundation, without receiving any assistance from external sources. And as it was entirely raised upon this foundation, the commencement of the building (to carry out our metaphor) must unquestionably be dated from the time when it was thus established, although the progress of the work was for a long time concealed beneath the ground, and although the courses first laid were imperfectly fixed, and had to be removed and replaced by others.

Such being our view of the merits of this essay, it seems to us that Mr. Shaw has underrated them, in keeping back, as he has done, those parts of it which show that Sir C. Bell made in the first instance an imperfect and erroneous application of his own principle. These do not appear to our minds to derogate in any material degree from his claims as a discoverer; and, by partially suppressing them, he has thrown the worst imputation on an individual, who, with all his faults, is scarcely capable, we trust, of the misrepresentation with which he is thus virtually charged. Let us see how the case stands.

The only experiment on the roots of the nerves recorded in this essay is the following:

"On laying bare the roots of the spinal nerves, I found that I could cut across the posterior fasciculus of nerves, which took its origin from the posterior portion of the spinal marrow, without convulsing the muscles of the back; but that, on touching the anterior fasciculus with the point of a knife, the muscles of the back were immediately convulsed. Such were my reasons for concluding that the cerebrum and the cerebellum were parts distinct in function, and that every nerve possessing a double function obtained that by having a double root." (p. 51.)

Now, *were this all* that is said on the subject, Mr. Shaw would be perfectly justified in saying, as he has done, that, whilst Sir C. Bell affirmed in sufficiently positive terms, that the anterior root was alone capable of exciting the muscles to contraction, he has refrained from saying directly to which root sensation belongs (p. 44). But we find passages in the essay, which show that he had formed an opinion on the subject; and these we feel it our duty to quote in full, slightly varying their order, that we may exhibit more clearly their bearing on one another.

"The cerebrum I consider as the grand organ by which the mind is united to the body. Into it all the nerves from the external organs of the senses enter, and from it all the nerves which are the agents of the will pass out.

"The nerves proceeding from the crus cerebelli go everywhere (in seeming union with those from the crus cerebri); they unite the body together, and control the actions of the bodily frame, and especially govern the operation of the viscera necessary to the continuance of life." (p. 54.)

Of these two classes of nerves an illustration had been previously given in the eighth and ninth pairs. "The first is a nerve of the class called vital nerves, controlling secretly the actions of the body; the last is the motor nerve of the tongue, and is an instrument of volition." The functions of the two central organs being thus assigned, the anatomical statements of their relations previously given would leave no room for doubt that similar functions would be assigned to the anterior and posterior roots of the spinal nerves, but the author has himself deduced the inference.

"From the medullary matter of the hemispheres there pass down, converging to the crura, striæ, which is the medullary matter taking upon it the character of a nerve; for from the crura cerebri, or its prolongation in the anterior fasciculi of the spinal marrow, go off the nerves of motion. But *with these nerves of motion*, which are passing outward, there are nerves going inwards, nerves of touch, and nerves of peculiar sensibility, having their seat in the body or viscera. It is not improbable that the tracts of cineritious matter which we observe in the course of the medullary matter of the brain, are the seat of such peculiar sensibilities, the organs of certain powers which seem resident in the body." (p. 56.)

"The secret operations of the bodily frame, and the connexions which unite the parts of the body into a system, are through the cerebellum and nerves proceeding from it." (p. 60.)

Now it is a little curious that, whilst these early ideas of Sir C. Bell's on the functions of the cerebrum and cerebellum should so nearly correspond with the present state of opinion respecting them, he should have been so greatly misled by his erroneous view of their anatomical relations, in assigning corresponding functions to the anterior and posterior fasciculi of the spinal cord. It is evident that he arrived at these conclusions by reasoning on the supposed termination of the cranial nerves in these organs, and that he interpreted the uncertain result of his experiment by the views he had thus formed. We assert, then, that Mr. Mayo was *not* guilty of the misrepresentation imputed to him by Mr. Shaw, in stating, as he has done,* that "Sir C. Bell was carried by these experiments very near to the truth, but he failed at that time to ascertain it; he inferred from his experiments, indeed, that the anterior and posterior roots of the spinal nerves have different functions, but in the nature of these functions he was mistaken. Upon the anterior root he supposed both motion and sensation to depend: the posterior root he considered an unconscious nerve, the office of which was to control the growth and sympathies of parts." On the contrary, we assert, that Mr. Shaw has been guilty of a positive but no doubt unintentional *suppressio veri*, in omitting all reference to the passages we have quoted, when professing to give a full exposition of Sir C. Bell's early views, for the purpose of showing their inconsistency with Mr. Mayo's statement.

But, in the sentence immediately following the passage just quoted from Mr. Mayo's Physiology, we find the following assertion: "*Before Sir C. Bell published any other account of the functions of these nerves, M. Magendie had given to the world the true theory of their uses.*" This is *verbally* but not *actually* true, as the course of our history will now fully prove.

Experiments on the roots of the spinal nerves alone are not likely ever to lead to satisfactory results, especially in regard to their *sensorial* character. The causes of the difficulty, originating in the violent nature of the operations concerned, are fully explained in Mr. Shaw's work, (pp. 44-6); and it is evident that Sir C. Bell was strongly impressed by them, for he abandoned this mode of enquiry for one which promised more certain information, whilst still pursued *on the same principle.* We have already noticed the contrast which he drew between the very *regular* distribution of the spinal nerves, and the very *irregular* course of the cranial; and it has been seen that he attributed the latter to a difference in their characters, corresponding with that which be believed to exist between the two roots of the spinal nerves. Nothing was so natural, then, as his recourse to the cranial nerves for the determination of the question which he could not solve by experiment on the roots of the spinal; and that this was the plan he followed, we learn not only from his own history (prefixed to his memoirs), but from the papers published during the course of the investigation by his brother-in-law and assistant, the late Mr. John Shaw, to which we shall have frequent occasion to refer.

The experiment on the spinal nerves had given satisfactory indication that the anterior column of the spinal cord is the portion specially connected with *motion.* Following it up to the crus cerebri, it was perceived that the ninth, sixth, and third nerves, arise by single roots from it. These were well known to be purely *muscular* nerves in their distribution; and the belief thence arose, that the anterior fasciculi of the spinal cord were not only peculiarly but *exclusively motor.* "The

* Outlines of Physiology, fourth edition, p. 249.

inference then presented itself," says Sir C. Bell, "that the posterior column and the posterior roots were for sensibility. But here a difficulty arose. An opinion prevailed that ganglions were intended to cut off sensation; and every one of these nerves, which I supposed were the instruments of sensation, have ganglions upon their roots. Some very decided experiments were necessary to overturn this dogma." Thus arose the series of experiments on the fifth pair, and portio dura of the seventh, which not only led to the establishment of the true functions of the roots of the spinal nerves, but also contributed towards the doctrine of the respiratory system.

But before entering upon these, we may observe, that the passage just quoted harmonizes fully with what we have stated of Sir C. Bell's early opinions. A vague notion was prevalent, that the posterior roots were connected with the "Vital and involuntary motions," in which sensation did not participate; and that the cerebellum, which was supposed to be their anatomical centre, was also the centre of these actions. Now, in the absence of any decided experimental results, it is quite evident to us, that Sir C. Bell did not at first feel justified in attacking this opinion, but acquiesced in it. It is not, therefore, a fair representation of his views, to say, that he drew such an inference from experiment; the fact being rather, that his first experiments did not contradict it. But the first which he performed on the cranial nerves were especially directed to the overthrow of the dogma, which impeded the general reception of the inference at which his own mind had arrived.

Without entering into minute historical details, we shall enquire what were the opinions of Sir C. Bell on the functions of the fifth and seventh nerves, as expressed either by himself or by Mr. J. Shaw previously to the publication of Mr. Mayo's *first* Series of Anatomical and Physiological Commentaries, in August, 1822. The sources from which we gather them are Sir C. Bell's first Memoir on the Nerves, read before the Royal Society on the 12th of July, 1821; Mr. J. Shaw's Manual of Anatomy, published in September, 1821; an Essay, by the latter gentleman, "On the Difference in the Functions of the Nerves of the Face," in the Quarterly Journal of Science, December, 1821; another by the same, "On the Effects produced on the Human Countenance by Paralysis of the different systems of Facial Nerves, April, 1822;" and a paper, by the same, on "Partial Paralysis," in the Medico-Chirurgical Transactions for April, 1822. No one, we believe, who is acquainted with the circumstances of the case, will hesitate in regarding Mr. J. Shaw as Sir C. Bell's mouthpiece at this period, and in assigning to the latter all the opinions on the subject of the nerves put forth at intervals by the former. We deem it right to quote Sir C. Bell's own reason for the form in which the results of his enquiries were first presented to the public.

"In proceeding to give some account of these new observations, the author of this paper had conceived that it would be more suitable to the scientific body he had the honour to address, to lay the subject before them in the precise form in which it first presented itself to his enquiries, and to detail his observations and experiments in the order in which they were made; but he has been persuaded by some of the members of this Society to change the form, and to present the subject in the manner to which he was accustomed in teaching these doctrines. They were pleased to say, that in this way a new subject would be more readily comprehended."

In a note to the present edition, the author adds:

"I believe that attention could not be raised to this great subject by the account of a system founded on anatomy, and on the minute distinctions in the origins of the nerves. It required the announcement of some distinct and remarkable facts, such as this paper contains, to excite enquiry." (p. 34.)

The adoption of this plan we believe to have been the source of much misconception; the author having, in his desire to direct attention to some prominent and novel points, left others unnoticed, and thereby given a handle to his opponents for the assertion that he was unaware of them. Thus he characterized the portio dura of the seventh pair as a "respiratory" nerve; and if he did regard it in any other light, he certainly did not so express himself. He almost passed by the question of the roots of the spinal nerves, referring to it only by the way; and it has thence been asserted that he left it as it lay after the first statement of his views on the subject. This is the very opposite of truth, as we shall now demonstrate.

The first point we meet with is the recognition of the analogy between the fifth pair and the spinal nerves. This, to be sure, is not new, since Soemmering and other anatomists had advanced the same doctrine. But it happens that it is very important to our argument to show that Sir C. Bell did not only recognize it at this time, but proceeded on the strength of it. For, if this be the case, we may safely assert that he regarded as true of the two roots of the spinal nerves that which he concluded from observation and experiment to be true of the two roots of the fifth pair. The double origin, the existence of a ganglion on one of the roots, the distribution both to muscles and integuments, and the connexion with the functions both of sensibility and voluntary motion, are dwelt on by Sir C. Bell and Mr. Shaw in support of this analogy; as also the similar connexion which they possess with the sympathetic. The anatomical description, however, of the origin of the fifth pair is very imperfect. Sir C. Bell states that "it receives roots both from the medullary process of the cerebrum and of the cerebellum. A ganglion is formed upon it near its origin, though some of its filaments pass on without entering the ganglion. Before passing out of the skull, the nerve splits into three great divisions, which are sent to the face, jaws, and tongue. Its branches go minutely into the skin, and enter into *all* the muscles, and they are especially profuse to the muscles which move the lips upon the teeth." It is evident, therefore, that Sir C. Bell was ignorant of the previous statements of Paletta, Soemmering, and Prochaska relative to the exclusive distribution of the ganglionless root to the muscles of the lower jaw; had he been acquainted with them, he would have been saved from the only error he committed in assigning the functions of the fifth pair.

In all the papers we have referred to, the fifth pair is spoken of as the exclusive source of the *sensibility* of the face. Although it is nowhere pointedly stated, it is evident from the contrast repeatedly drawn between the fifth and seventh nerves, that Sir C. Bell considered the former to possess a sensory character in virtue of its ganglionic root.

Of this we do not entertain the smallest doubt; and we think that no candid person who examines the evidence can avoid arriving at the same conclusion. Remembering, then, the analogy drawn by him between the fifth and spinal nerves,—bearing in mind the continuity which he believed to exist in the tracts from which these two roots respectively originated,—and the fact that he had recourse to the cranial nerves for the special purpose of clearing up what was left obscure in the spinal,—are we not justified in asserting that he thus *virtually* assigned sensibility as the function of the posterior roots of the spinal nerves? If this be admitted, it follows that the claim of M. Magendie, and of Mr. Mayo in his behalf, falls to the ground; since the experiments of the former were not published until August 1822.

If any doubt could remain on this subject, it is removed by Mr. Shaw's paper in the Medico-Chirurgical Transactions already referred to, and also by a statement contained in his Manual of Anatomy. Here the doctrine that the two origins of the spinal nerves are subservient to motion and sensation respectively is *most distinctly implied;* but, curiously enough, it is not pointedly expressed. On this subject our readers shall judge for themselves:

"I shall take the liberty of trespassing still more upon the time of the Society, by making a few remarks upon a very curious question, which has particularly excited the attention of physicians in all ages since the time of Galen. 'Why sensation should remain entire in a limb, when all voluntary power over the action of its muscles is lost, or why muscular power should remain when feeling is gone?' The attention of Galen was particularly directed to this question, in consequence of his having been called on by his contemporaries to account for the manner in which he had cured a paralysis of the finger, by applications made to the spine. In answer, Galen told them, that two sets of nerves went to every part; one, to endow the skin with sensibility, the other, to give the muscles the power of voluntary action. This opinion was probably founded on an erroneous theory; but the facts lately discovered, and the observations which have been noted in attending to the phenomena of disease, though they do not afford absolute proofs of the correctness of Galen's supposition, still go far to establish the fact, that every part of the body which is endowed with two or more powers is provided with a distinct nerve for each function.

"The form of the nerves, which at the same time endow the skin with sensibility, and the muscles with the power of voluntary motion, is such, that they appear to be single cords; but, if we examine the origin of any of these nerves, we shall find that it is composed of two packets of fibres, which arise from distinct parts of the spinal marrow. These origins are soon enveloped in the same sheath, so as to appear to a superficial observer to form a single nerve. It is not too much to suppose, that either of these origins may be affected while the other remains entire. To prove this by ocular demonstration will perhaps be impossible; and, therefore, the question will probably remain undecided. But we have already seen examples of the consequences of injury to a nerve that has a single root, viz., the portio dura; for, if we cut it, there will be only one set of actions paralysed, while, by dividing a nerve which has a double origin, viz., the fifth, we shall destroy two powers, namely, voluntary motion and sensibility. We know, also, that when we cut through the trunk of a nerve going to the hand, we destroy both sensibility and voluntary motion." (*Shaw's History*, p. 13.)

Mr. Shaw then refers to the early experiments of Sir C. Bell on the spinal nerves, and states the uncertainty in which they left the question of the source of their sensibility. But, it being remembered that the object of his Essay was to explain the functional differences of the fifth and seventh nerves in accordance with their origins, he evidently implies that the doubt is in his mind cleared up, and he thus concludes:

"If the view which I have here taken of this question be correct, it may lead to this rule of practice. If only one set of functions of a spinal nerve be deficient, we should apply our remedies to that part of the system from which the nerve arises; but if both functions are impaired, we must then direct our enquiries to the state of the nerve in the whole course from its origin to its distribution, as the loss of power is probably owing to some affection of a part of the nerve, after the two sets of filaments by which it arises are united together." (p. 15.)

In his Manual of Anatomy, Mr. J. Shaw was still more precise as to the functions of the respective roots of the spinal nerves, and that his meaning was understood is evident from the following account of his views given by Magendie in his Journ. de Physiol. Oct. 1821: "Every nerve that belongs to the regular system arises by two separate roots, one from the anterior and one from the posterior column. . . . . There are thirty-two perfect, regular, and double nerves, common to man and the lowest animals, and each possessing motion on the one hand, and sensation on the other."

In concluding, then, this part of our history, we confidently assert that, at the period of the publication of his first memoir, Sir C. Bell had abandoned the notion of the functions of the roots of the spinal nerves with which he started in 1811; and that he had arrived at the conclusion, that the *anterior* roots of the spinal nerves and the small root of the fifth pair are *motor* in their function, and the *posterior* sensory. To any one who still objects that this *change is nowhere expressed,* we would only speak thus: The first essay not having been *published,* Sir C. Bell did not feel it incumbent on him to avow any change, however prudent it might have been to do so. He may still fairly refer to it for the enunciation of *his principle,* which has undergone *no change.* The whole internal evidence supports the view that he had by this time formed the opinion of the sensory character of the posterior roots of the spinal nerves. On the contrary, let any one read the papers we have referred to, with the opinion that the posterior roots of the spinal nerves had in Sir C. Bell's estimation *any other function than sensibility,* and he will be constantly unable to harmonise it with what he there encounters. A useful lesson may hence be learned by those who are desirous of making known their discoveries and securing their title to them. Let the previous knowledge on the subject be fully ascertained and detailed, and the supposed improvements systematically enunciated, without leaving anything open to doubt, excepting such points as are intentionally reserved for future examination. Had Sir C. Bell done this, instead of yielding to the wishes of his friends, much angry discussion would have been saved.

In regard to the functions of the *fifth pair,* no one who reads Sir C. Bell's original memoir can feel the smallest doubt that its author entertained erroneous notions. His ignorance of the minute anatomy of the roots led him to believe that, as in the case of the spinal nerves, the motor and sensory branches accompanied each other throughout. He accordingly speaks of the nerve as supplying all the muscles of the face with motor influence, as well as the surface with sensibility. But it is clear that he regarded the action of mastication as the *special* object of its motor branches, since he speaks of the whole as the nerve of *sensation and mastication.* He was aware, too, that the supra-orbital branch does not possess motor powers, since he speaks of its having been divided, so as to cause loss of sensibility, without the movements of the eyebrow being affected. But *these*, as will presently appear, he regarded as respiratory or expressional in their character, and therefore in the province of the portio dura. That it was the only nerve of sensation belonging to the face, he adduced abundant proof. But in regard to its motor properties the experimental proof was incomplete. The only fact of this kind which he brought forward was the very one which induced him to uphold the opinion he had formed that the motor filaments are distributed to the whole face, being the experiment upon the infra-orbital branch in an ass, section of which *appeared* to deprive the animal of the power of moving his lip. The fallacy of this inference was subsequently exposed by Magendie and Mayo; and Sir C. Bell, as we shall see, has adopted the correction without due reference to the source from which it was supplied. We think there is good reason to believe, however, that, by the union of anatomical and experimental enquiry, Mr. J. Shaw would have speedily arrived at the truth by his own investigations. That the fifth pair had motor properties, however, was abundantly proved in Sir C. Bell's estimation by the origin of its smaller root from the *tractus motorius*, and by the persistence of some amount of motor power after complete paralysis of the seventh. So far as this relates to the masticator muscles, there can be no mistake; but we suspect that Sir C. Bell was at first confirmed in his idea of the *general motor* character of the fifth by the occurrence of cases in which the movements of respiration and expression are paralysed, while the voluntary power over the muscles remains. The latter we do not think he was *then* prepared to attribute to the seventh pair.

Everything in the original memoir leads us to the conclusion, that Sir C. Bell *then* regarded the seventh pair as solely concerned in the *respiratory* movements of the face. These, however, he made out to be a very comprehensive class, embracing not only the motions of expression, but many actions which are usually regarded as voluntary. It is quite certain that *no other* motor functions were there attributed to it; and there is an antagonism evidently alluded to (*Phil. Trans.*, p. 422) between the voluntary action of the fifth nerve, and the involuntary action of the seventh. Perhaps the strongest proof, however, that we are correctly stating Sir C. Bell's original opinion, is the alteration which all the passages have undergone in which the fifth pair is spoken of as the general nerve of voluntary motion, and the seventh, either directly or indirectly, characterized as simply a respiratory nerve. If he has not altered his opinions on the subject, why has he thought it necessary to alter so completely the expression of them? We are sorry to be obliged to add, that he has not marked these altered passages as he should have done; so that the reader of the last edition will not find, in the paper of 1821, Sir C. Bell's *original* opinions or experiments, but modifications of them rendered necessary by the subsequent progress of his views. There could be no possible objection to his ingrafting these on his original memoir if he thought fit; but there should have been some intimation given of a change. We can very well imagine a student referring to this paper for the purpose of ascertaining the original opinions of Sir C. Bell, and being entirely deceived as to the degree of truth he had then attained on the points in dispute. The charge of unfair dealing has been raised against him by the editor of the Dates and Documents; and a considerable amount of evidence might be adduced in support of such a charge. We shall content ourselves with stating the fact, in the hope that Sir C. B. may see the propriety of not giving such a handle to his opponents, as is afforded by what certainly has the *appearance* of underhand dealing.

There is another curious point to which no one, we believe, has directed the attention of the public. In his original memoir (p. 411), Sir C. Bell speaks of the seventh nerve as distributed not only to the muscles, but *to the skin*, in conjunction with the minute vessels of the cheek. And in another passage (p. 415), he speaks of *an impression on the respiratory nerves*, in such a manner as to lead to the belief that he regarded the seventh pair as not only an efferent or motor nerve, but, so far as the function of respiration is concerned, an afferent or excitor channel. If he entertained this opinion, (which, however, we do not positively state,) though it receives strong confirmation from an expression of Mr. Shaw's, he soon abandoned it, for we find the anatomical assertion first qualified and then omitted, and the other expression modified. Whether or not this idea be correct, (and, like the other, it is borne out by the alterations which the author has made,) it derives support from the fact that the anatomical statement does not correspond with that of other authors, nor with that of Mr. Charles Bell in his Anatomy of the Human Body, (1802, vol. iii. p. 150-1,) where all that is said of its ultimate distribution refers to muscles only. A passage from the work just referred to may be thought a little curious by some of our readers. "As to the sympathies which physicians have thought fit to ascribe to the connexions of this with other nerves, as laughing, weeping, kissing, &c., they would be tedious to enumerate, and by no means instructive."

Mr. A. Shaw endeavours to show from his brother's papers, that, previously to Mr. Mayo's first publication, the *voluntary* character of the portio dura was understood by Sir C. Bell; but we do not think that he has succeded. He adduces its known concern in the actions of whistling, speaking, blowing, and inhaling, as proved by cases of paralysis; but all these actions belong to Sir C. Bell's class of *respiratory* movements, and are expressly alluded to by him as such; and in his eagerness to establish this entirely new system, he seems to have left out of view the share which volition has in producing them. The only action which could be otherwise represented is the closure of the eyelids, of which Mr. J. Shaw observed that a patient with paralysis of the seventh nerve was incapable. But even this Sir C. Bell tried to include in his class of respiratory movements; for in the first reprint of his Memoir, in 1824, he says, in relation to a case of this kind, "*Thus it appears* that the portio dura of the seventh nerve is the principal muscular nerve of the face; that it supplies the muscles of the cheek, the lips, the nostrils, and the eyelids; *that is,* that it is the nerve which orders *all those actions which are in the remotest connexion with the act of respiration.* It is possible that those relations may not be apparent at first; but in the prosecution of the subject we shall discover the reasons of those links by which the respiratory organs are combined with the actions of the features."

We now come to the period when other physiologists present themselves as participators in the work of discovery. In August, 1822, M. Magendie published his first Memoir on the spinal nerves, in which he assigned distinct functions to the two roots. Now we have stated our full conviction that Sir C. Bell and Mr. J. Shaw had virtually done this in the year previously, and that it was so understood at the time. Moreover, it is quite clear that M. Magendie was well informed, through various sources, of what had been previously done in England. For details on this subject we must refer to Mr. A. Shaw's history; only stating that among these sources was a visit paid to M. Magendie by Mr. J. Shaw himself in the autumn of 1821; at which time the latter explained Sir C. Bell's views, performed in his presence experiments illustrating the functional difference of the fifth and seventh pairs of nerves, and presented him with a copy of his Manual of Anatomy, in which a sketch of these views is given. All this is acknowledged by M. Magendie. It would be impossible, then, to sustain the slightest claim to priority in discovery in his behalf, even if the doctrines he aimed at establishing were the same.

But they are not so. Even in his first Memoir (Aug. 1822), he hints at *a union of the two functions in each root.* His experiments, he states, "made it appear that the posterior roots were *more particularly* provided for sensation, while the anterior were *more particularly* provided for motion." In the next paper (Oct. 1823), he expresses this opinion decidedly, and he has continued to hold it to the present time. He can scarcely, then, be said to have made any advance upon Bell's original hypothesis; and it is preposterous to set up any claim to discovery, either of principle or of fact, in his behalf. Into the merits of his opinion we shall not enter at present. No one, we apprehend, will attach great weight to it; since his deficiency of almost every habit that characterizes the true philosopher renders him nearly incapable of correct reasoning on any complex subject. We again repeat that the true functions of the roots of the spinal nerves are learned, in by far the most unexceptionable manner, from experiments on the cranial; and these M. Magendie has almost entirely neglected.

On one point, however, he did make an advance towards the truth. It was he who first detected the imperfection of the experiments which led Sir C. Bell to attribute motor functions to the infra-orbital branch of the fifth pair; showing that section of it does not destroy the motor power of the lip. But, in a subsequent paper, he stated that the seventh nerve is possessed of sensibility; an error of great importance in relation to the principle of Bell that single roots give functions of a single class only; and this was exposed by Mr. J. Shaw, who showed that the supposed sensibility of this nerve is derived from filaments of the fifth pair. Moreover, he never intimated the opinion that the fifth pair has *any* motor power, nor did he pay the slightest attention to its double origin; but he everywhere alludes to its sensory properties only; so that it may reasonably be questioned if he knew anything more respecting it. His strange idea regarding its participation in the functions of special sensation is well known, and valued as it deserves.

We now come to a more painful division of our history;—that which respects Mr. Mayo's claim. We cannot but deeply regret the position he has assumed in this question. He has not merely arrogated to himself, as we shall presently see, discoveries to which he has no right whatever, but he has supported his claim by unwarrantable means; and, influenced apparently by personal hostility to Sir C. Bell, he has attempted to deprive him in favour of Magendie of that to which he could not assert his own right. All this would be pitiful only in a man of no other pretensions to eminence; but it is something worse in one who has such well-earned and independent claims to notice.

Before proceeding to these particulars, we think it right to state generally (on the authority of Mr. A. Shaw) the relation which formerly subsisted between Sir C. Bell and Mr. Mayo. The latter was not merely an attendant at the Windmill-street School, where Sir C. Bell's peculiar opinions were made the subject of frequent prelection by himself and Mr. Shaw, but he was Sir C. Bell's house-pupil, and therefore enjoyed special opportunities of becoming acquainted with the progress of his opinions. Moreover, he had been employed by Sir C. Bell in making preparations illustrative of the difference between the roots of the cerebral nerves. Further, he had given other assistance to Sir C. Bell and Mr. Shaw, which was duly acknowledged at the time in three of the papers we have referred to. We quite agree with Mr. A. Shaw in attributing to these circumstances the degree of credence which his statements have received.

"It cannot be questioned that it has been owing to this gentleman's having been originally a pupil of Sir Charles Bell, and to its being generally known that he enjoyed, on that account, the best opportunities of knowing the true history of the discoveries, and likewise, I may add, to its being commonly felt that, unless he had been strongly assured of the correctness of what he delivered, he would have been unwilling to pass an unfavorable judgment concerning his teacher,—that his representations, however unsupported by proofs or arguments, have been readily accepted by the profession as infallible." (p. 24.)

The first part of Mr. Mayo's Anatomical and Physiological Commentaries was published in August, 1822. In *this* he took up a position decidedly hostile to Sir C. Bell, not on one or two points only, but as to *the whole system.* The title of the paper in question was "Experiments to determine the Influence of the Portio Dura of the Seventh, and of the Facial Branches of the Fifth Pair of Nerves." It might have been expected that he would have adopted the principles of his master; and, by additional experiments, have corrected or confirmed the functional details which had been imperfectly ascertained. On the contrary, he not only *neglects* but *opposes* the principle throughout. In giving a minute description of the anatomy of these two nerves, he omits all reference to the difference of their origins; but commences his account of their branches from the time of their emergence from the cranium. Moreover, he says that the "only unexceptionable evidence" respecting the functions of individual nerves is derived from the results of injuries

(accidentally or purposely inflicted) in their course. This is in evident opposition to Sir C. Bell's principle, which has been fully explained by Mr. J. Shaw in his paper on Partial Paralysis, which had been read to the Medico-Chirurgical Society five months before. But Mr. Mayo offers even more decided opposition to it than this. He quotes a passage from Sir C. Bell's memoir, in which the fifth pair is classed with the spinal nerves on account of its double origin, ganglion on one root, universal distribution,&c.—and the portio dura with the respiratory nerves, on account of their single ganglionless roots and peculiar distribution; and says of it "*As nothing is so prejudicial to the interests of science as the temporary adoption of an unsound theory*, I shall hazard a few remarks upon that of Mr. Bell."

It is evident, then, that Mr. Mayo cannot found on this paper the slightest claim to having assisted Sir C. Bell in the development of his *principle*. We shall now enquire what is the amount of its contribution to positive knowledge. In the first place, with the view of demolishing the "respiratory system," he performed experiments on the *par vagum*, for the purpose of demonstrating that it is a *common nerve* of motion and sensation. Some of these experiments were certainly important; but they by no means supported the inference erected upon them; nor have they, as is very well known, prevented the necessity for others of more varied and precise character. He then endeavours to prove that the portio dura is "*a common nerve of voluntary motion;*" if it be divided, the muscles which receive branches from it are "*completely paralyzed.*" Here he supplies a correction to Sir C. Bell, which was, we think, partly needed. The latter had, we feel no doubt, overlooked in the first instance the participation of the seventh pair in the *purely voluntary* motions of the face; and had endeavoured to show that all the movements it produces are connected remotely or directly with the act of respiration. But Mr. Mayo runs into the opposite extreme; for he asserts that this nerve is not merely the channel of *voluntary motion only*, which is certainly untrue, but that it supplies *the whole* of the voluntary power of the nerves to which it is distributed—a position still more decidedly false. This brings us to consider his statements regarding the *fifth pair*.

Neglecting, as we have stated, all consideration of the peculiar origin of this nerve, and, as it would seem, desiring nothing so much as to prove the fallacy of Sir C. Bell's statements, Mr. Mayo confined his experiments to the particular subject of Sir C. Bell's, namely, the infra-orbital branch: and succeeded in proving that muscular power had been erroneously attributed to it. But in this correction he had been anticipated, as we have seen, by Magendie, who had published the same correction ten months before. Mr. Mayo is represented by Mr. A. Shaw as leaving the question in this state, and as implying, if not asserting, that the fifth pair has no other function than sensibility. But this is not the case, as the following passage shows:

"I infer from the preceding experiments that in the ass the portio dura is a simple nerve of voluntary motion; and that the frontal, infra-orbitar, and inferior maxillary, are nerves of sensation only; and from the preceding anatomical details, that other branches of the third division of the fifth are voluntary nerves to the pteregoid, the temporal, and buccinator muscles." (*Commentaries*, p. 112.)

The concluding passage is a good specimen of the *animus* which characterizes the whole paper.

"It remains (says Mr. Mayo) for the reader to decide whether Mr. Bell's experiments are satisfactory, and bear out his inferences; whether the latter, coupled with my former observations on the five respiratory nerves of this author, leave his theory tenable; and, perhaps, finally to determine whether there exist in the whole of Mr. Bell's essay, after the deduction of his controvertible statements, *more than one correct inference.* I here allude to Mr. Bell's experimental confirmation of an opinion which, at the beginning of the eighteenth century, occurred to Dr. Blair, on his minute examination of the proboscis of an elephant, viz., that the infra-orbital nerves are nerves of touch."

Within two months after the publication of Mr. Mayo's first paper, Mr. J. Shaw obtained unequivocal proof, by experiment, that the *third branch* of the fifth pair has motor properties, and that it is the nerve which produces the masticator movements of the jaws. This he published in a paper entitled "Observations on M. Magendie's Experiments," in the London Medical and Physical Journal, for October, 1822. Thus, then, we may regard the *functional history* of this nerve as completed. We do not perceive, however, that any attempt was made by Sir C. Bell to obtain more accurate information regarding its origin; but if he had been aware of the description given of its roots by previous anatomists, he must at once have seen how perfectly conformable it was to the results obtained by experiment and observation. Throughout the paper we have just referred to, *Magendie's* experiments are the subject of criticism. The correctness of his statement respecting the absence of motor power in the infra-orbital branch is admitted; and the fallacy of the first inference explained. The paper concludes with a notice of "a late pamphlet," which Mr. Shaw adverts to with much unwillingness; "for, when engaged in such a delightful enquiry, it is most unpleasant to be involved in a dispute with one whose object seems rather to be an attempt to detract from the merit of his late master than a wish to promote the interests of science." It is perfectly evident, then, that Mr. Mayo has *no right whatever* to claim, as he has always done, the correction of Sir C. Bell's views regarding the fifth pair; and that no acknowledgment was due from Sir C. Bell *to him* in adopting the modified opinion; but the obligation to Magendie should have been more openly stated. We find it only in the *second* memoir; whilst the correction has been introduced into the reprints of the first paper, without any notice of the alteration.

We shall quote two other passages from this and the succeeding paper of Mr. Shaw, in confirmation of views we have previously expressed. The first will show *his* anxiety that Sir C. Bell's doctrine regarding the *spinal nerves* should be the first in order of publication: "The accumulation of facts will probably embolden Mr. Bell, in a short time, to give an account of his investigations into the anatomy of the spinal marrow, and the views he has deduced from them, which will lead to an entirely new doctrine of the anatomy of the brain and nerves. In justice to Mr. Bell, I should state that this has been a subject of constant contemplation with him for the last fifteen years. *He has thought it necessary to rouse attention to the subject* of the nerves, by first offering his papers on the respiratory nerves, and on those of the orbit and throat, before that on the general system. It has been in vain that we have urged the danger of anticipation."*

In the first part of Mr. J. Shaw's General View of the Nervous System, in the same journal for December, 1822, we find the following passage, which confirms our opinion that the actions of the portio dura were still regarded as of a respiratory character *only:* "The observations which are already before the public, proving that the expression of the face, nay, of the whole body, has such an intimate connexion with the organs of respiration, afford sufficient explanation why the same nerve should combine the *motions of the eyelids and forehead* with the acts of respiration, and with the motions of expression performed "through the muscles of the nose and mouth."† And he speaks in the same paper of the portio dura as being a nerve "*as similar in function as it is in origin and form* to the branches of the *eighth pair.*" This last sentence also tallies with the passage in Sir C. Bell's memoir formerly alluded to as indicating a belief that the portio dura is subservient to *impressions* connected with respiration, as well as to motor influence; since the par vagum has been known to have chiefly an afferent function from the time of Whytt. Neither Mr. J. Shaw nor Sir C. Bell have ever acknowledged any change in their views on this subject; but the question again returns—if their views have not been altered, why has the mode of expressing them been gradually changed ? We do not think, however, that Mr. Mayo can claim much merit on this score; since the influence of the will may be distinguished from an instinctive impulse more by cases of paralysis in man, in which one function is lost without the other, than by any experiments that he had performed or could possibly devise.

Previously to the publication of the second part of Mr. Mayo's Researches, in July, 1823, two new memoirs had been presented by Sir C. Bell, to the Royal Society; one on the Nerves of the Orbit, and the other on the Motions of the Eye. These, however, do not contain any matter peculiarly relevant to the present enquiry, which refers only to the general functions of the nervous system, and the offices of the fifth and seventh nerves: the latter question being connected, as we have seen, most closely with the first. The chapter of Mr. Shaw's History devoted to the consideration of Mr. Mayo's second paper is thus headed: "Mr. Mayo's second memoir essentially distinct from his first, inasmuch as he abandoned his opposition to the principle of the discoveries, and adopted it for his own use, without acknowledgment." This appears fully justified by the contents of the paper.

We have seen that, in his first memoir, Mr. M. opposed the principle we have so often referred to as peculiar to Sir C. Bell, that the explanation of the functions of nerves is to be sought in their roots; and that, in his anatomical description, he omitted all mention of the origin of the fifth pair.

He *now* adverts expressly to its roots, and speaks of it as *well known* that the small root passes by the ganglion and enters the third division only of the fifth pair. He then informs us that he had discovered that this is distributed exclusively to the muscles of the jaws; and, reminding

* London Medical and Physical Journal, xlviii., p. 347.
† Op. cit., p. 463.

us that the remaining branches had been proved to be nerves of sensation, he continues, "*Thus it appears* that the fifth nerve consists of two portions: one of which has no ganglion, and is a nerve of voluntary motion, and probably of muscular sensation; and another, which passes through a ganglion, and furnishes branches which are exclusively nerves of the special senses." He then quotes Soemmering as having long ago pointed out the analogy between the fifth pair and the spinal nerves; and modestly tells us that "by this analogy he was led to conjecture the fact that the double roots of the spinal nerves have functions corresponding with those of the fifth," and that, "while he was engaged on experiments to ascertain the fact, M. Magendie's were published, which established the justice of the conjecture."

We scarcely know how we can decorously characterize the latter part of this claim. Suppose Mr. Mayo had been the discoverer of the accordance between the double function of the fifth pair and its double root, is it not perfectly clear that he had been led to this by the previous enquiries of Sir C. Bell, which had commenced with the spinal nerves, and afterwards been directed to the fifth as analogous to them, and as affording from its contrast with the seventh a more satisfactory object of comparative investigation? And is it not clear, also, that the functions of the roots of the spinal nerves had been correctly assigned two years before in Mr. Shaw's Manual, not to mention the assumption of them in Sir C. Bell's own memoir on the very ground specified by Mr. Mayo? It would have been *bad enough* for a bystander to have overlooked all this; but that Mr. Mayo, who had enjoyed such peculiar opportunities of knowing the real progress of Sir C. Bell's views, should have assumed that he was ignorant in June, 1822, of the true functions of the roots of the spinal nerves, we must say, is *too bad*. But this is not all.

Mr. Mayo claims the discovery of the anatomical connexion between the small root of the fifth pair and the muscles of the lower jaw; speaking, however, of the entrance of this root into the third branch exclusively as *well known*. Now we have before shown that the anatomists, who had stated the latter fact, which had been forgotten and neglected, also knew the former. How much credit, then, does Mr. Mayo deserve for this observation? We might have been disposed to award him a share, if he had not, in the same paragraph, passed by Sir C. Bell to give to Soemmering the analogy between the fifth pair and spinal nerves. But let it be clearly understood, that to Sir C. Bell is unequivocally due the first demonstration, that all the branches of the fifth are sensory, and that this is *the* nerve of sensation to the face; that after the correction of Magendie he had restricted its motor character to the third branch; and that Mr. J. Shaw had proved experimentally, which Mr. Mayo had not, that the third branch really does possess motor powers; and then let us ask, what share Mr. Mayo had in establishing *any one truth* regarding the functions of the fifth pair? Will it be credited that, in thus changing both his mode of enquiry and in adopting as the results of his own investigations the facts previously observed by Sir C. Bell and Mr. J. Shaw, he has not mentioned the name of either of these gentlemen from beginning to end of this paper, devoted as it is to enquiries connected with the roots of the cranial and spinal nerves?

We have yet to notice Mr. Mayo's Outlines of Physiology; but of these we shall soon dispose. We have already noticed that statement which refers to M. Magendie's claim to the discovery of the true functions of the spinal nerves, as *entirely destitute of foundation.* We say *the same* as to Mr. Mayo's claim to having established the motor character of the ganglionless portion of the fifth, and the true function of the seventh. And we would say, if possible, still more, as to the claim which he here repeats of having *been led by these discoveries* to assign the functions of the roots of the spinal nerves at the time of the publication of Magendie's experiments. We have yet a graver charge against him. He quotes from the *first part* of the Anatomical and Physiological Commentaries, the experiments which led him to the inference that the infra-orbital branch of the fifth is sensory only, (in which, as we have seen, he had been anticipated by Magendie, yet he gives the French physiologist no credit for anything *he* could claim;) and he then continues his account of experiments and observations on the same subject, embracing those contained in his *second* Memoir, in such a manner as would lead any one unacquainted with the facts to the idea that he was all along recapitulating the statements in the *first*. The difference of a year is here very important; and in the only other reference he makes to his Commentaries, he speaks of them as published in 1822, without intimating that a second part appeared at any subsequent time. The readers of his Physiology, therefore, have no information of the existence of such a publication; and naturally attribute to the *first* part all that is quoted from it. We can speak from experience as to the effect of this and other misrepresentations of Mr. Mayo's, in giving rise to most erroneous impressions as to the share which Magendie and himself had in the late discoveries; and there is one most important *omission*, with noticing which we shall conclude our reference to him. He leaves so little for Sir C. Bell to claim, that one would have thought he would have been glad to assign him what he could. But no; the same *animus* prevails throughout; and, in professing to award to each their due, he passes by the fact that "to Sir C. Bell we are indebted for introducing the new mode of iuvestigating the functions of the nerves, by examining the distinctions between their origins; and then indirectly claims that discovery for himself."

To sum up in few words the result of our historical enquiries, we may state that Sir C. Bell appears to us to have the exclusive merit of originating the mode of investigation which has led to the positive knowledge of the structural distinctness of the afferent and efferent nerves; that he was the first to give a correct explanation of the functions of the two roots of the spinal nerves; that he was the first to direct attention to the double function of the fifth nerve contrasted with the single function of the seventh, and to point out the connexion of the functions with the double and single roots of these nerves respectively; and that he was the first to assign clearly the whole function of the fifth pair. In thus speaking of Sir C. Bell, we include, of course, Mr. J. Shaw. We have no means of assigning to these gentlemen, individually, the particular share of merit which may be respectively due to them. Mr. J. Shaw always represented himself as following out Sir C. Bell's views; and Sir C. Bell has constantly taken opportunities of attributing to his regretted friend and relative the highest merits which such an assistant could possess. We wish that this spirit had been more extended to others who were engaged in the investigation.

The only points on which we regard Sir C. Bell as having received assistance or correction in perfecting the system as we at present possess it, are the character of the infra-orbital branch of the fifth, as a purely sensory nerve; and that of the portio dura of the seventh as a nerve of voluntary power, independently of its connexion with the respiratory function. For the first he was indebted to Magendie; and the obligation has been expressed by him, although, as we think, insufficiently. For the second he may possibly be indebted, in some degree, to Mr. Mayo; but as that gentleman's view is as erroneous in its exclusiveness as Sir C. Bell's own, we are disposed to think that the modification of the opinions of the latter is the result of his own independent observations. We shall presently see that it has been very gradual. As to the anatomical information respecting the roots of the fifth, which Mr. Mayo claims to have supplied, we have reason to believe that he was anticipated, not only by Paletta but by Mr. J. Shaw. The decisive result of the experiment of the latter upon the third branch, and the correction supplied by Magendie regarding the second, would naturally lead him to examine the anatomy of their roots with more precision; and we find from a subsequent statement of Sir C. Bell that this was the case, and that it was then perceived that the motor root is only connected with the infra-orbital branch by cellular substance.

With regard to the respiratory or superadded system of nerves, no one has ever attempted to deprive Sir C. Bell of his claim of originality. But the doctrine has not been by any means generally received among physiologists. That the respiratory movements belong to a large class of actions excited through the afferent nerves, central organs, and efferent nerves, was pointed out by Whytt, and has been generally taught in this country, except by those who adopted the strange notion that they are voluntary. It was very properly urged, therefore, by Dr. Alison, in opposition to the "respiratory system," that these movements are in no respect different in character from those to which no such system has been assigned. We fully accord with that philosophical physiologist in this view; and on a former occasion we entered at length into the analogy of the respiratory movements with those of deglutition, defecation, &c. (See Vol. V. p. 511, &c.) The tendency of Dr. M. Hall's researches has been to harmonize the two opinions on a very remarkable manner. He agrees with Dr. Alison and others who have written on the same side, in placing the respiratory movements in the same footing with those which have been generally denominated *sympathetic*; and he includes Sir C. Bell's respiratory system of nerves in his own distinct spinal system, to which all these associated movements may be alike referred.

We have purposely omitted all reference to the researches of Bellingeri on the Fifth and Seventh Pairs of Nerves, because there is no proof that, although they were contemporary with Sir C. Bell's, and were published before them, they contributed anything to the advance of knowledge in this country. But our summary would not be complete without a notice of them; and we should have had a still better opinion of Sir C. Bell's candour if he had mentioned them in his introductory history. These researches were published in an Inaugural Dissertation at Turin in 1818; but it does not seem that they received any attention in this country until 1834, when an abstract of them appeared in the Edinburgh Medical and Surgical Journal. The following statement we make on its authority: "The author sent a copy of the Dissertation to the Royal Society of London in 1819; and it was presented to that learned body on the 20th of January, 1820, as is proved by its being acknowledged at that date among the list of donations. There, however, it appears to have been buried in the inglorious oblivion of the multitude of works which find their way into that far-famed institution, or——but no, we cannot allow ourselves to give utterance to the alternative." Of that alternative we shall leave our readers to judge for themselves: we prefer not expressing an opinion of our own; but it is our duty to state facts.

Bellingeri first gives a minute anatomical description of the origin, course, and distribution of the fifth pair. He correctly describes its two roots, the formation of the ganglion upon the large one exclusively, and the entrance of the smaller one into the third branch alone. He states that few of the filaments of the first two branches are distributed to muscles, but that the third branch sends a larger proportion of filaments to muscles than either of the two others; and, from these muscles being those concerned in manducation, Bellingeri has given, with Paletta, the term nervus masticatorius to the smaller root. Amongst its branches he minutely describes the ramus buccinalis labialis, which Sir C. Bell took credit to himself in 1829* for having traced as the nerve governing those muscles of the lips which are concerned in mastication.

The portio dura of the seventh pair is described with equal minuteness. The chief peculiarity in the account of it is that, although the great majority are sent to muscles, some filaments are spoken of as proceeding *to the skin* of the cheek, lips, and neck. The correspondence of this with Sir C. Bell's early statement already noticed is somewhat curious.

We shall not enter into minute detail regarding his view of the functions of these two nerves, since a copious abstract of this part of his Dissertation is readily accessible elsewhere. The following is, we believe, a correct general description of them. Following the division of Bichat into the nervous system of animal life and that of organic life, he labours to show that the fifth pair belongs chiefly to the latter, controlling the growth and nutrition of parts, and being the medium of the *organic sympathies,* in which he includes not only what are now denominated such, but the sensible movements which take place without the control of the will, such as that of the iris, the internal auditory apparatus, &c. These he imagines not to be produced through the brain or spinal cord, but through the medium of nearer ganglia and plexuses. The motor powers of the third branch he shows to be connected with organic life, as ministering exclusively to the actions of nutrition. He attributes to the fifth pair the control over the secretions,

* Phil. Trans., Second Memoir on the Nerves of the Face.

both of the glands and membranes; and shows that it thus furnishes the *conditions necessary for the exercise* of the special sensory organs. In this he has anticipated very remarkably the correction which the subsequent dogmas of Magendie received.

Now we here find no trace of the function of sensibility attributed to the ganglionic division of the nerve by Sir C. Bell; and it cannot be denied that Bellingeri was so far carried away by his peculiar view of its character, as not to recognize this property. But it is equally clear that he has anticipated later discoveries in attributing to the fifth pair an influence on the functions of nutrition, secretion, &c., which it probably possesses in virtue of its ganglion. And the knowledge since attained of its character as an *excitor* nerve has enabled us to perceive that it does participate in many of the sympathetic movements he mentions, though not by directly acting on the muscles.

As to the portio dura, Bellingeri was nearer the truth. He distinctly states that it is the nerve of *voluntary motion* to the face, and also of the *animal sympathies.** He supports this statement upon experiments and pathological observations, which prove it as clearly as Sir C. Bell's. He was misled by the supposed distribution of the nerve to the skin, to consider it the nerve of sensation ; notwithstanding that, in a case he details, sensation is spoken of as unimpaired when the paralysis of the muscles supplied by the seventh pair was complete, its trunk being compressed by purulent matter in the aqueduct of Fallopius. We have already adverted to a somewhat corresponding idea on the part of Sir C. Bell. The following is from Bellingeri's general summary: "We conclude, therefore, that the facial nerve *performs almost all the voluntary motions in the head*, presides over animal sensation in the face and neck, produces laughter, *expresses the emotions of the mind and the passions of the soul*, and is the agent of all the animal sympathies." His notion that it was the channel of sensation appeared to derive support from experiment. He found that when it was divided on both sides, the animal gave no signs of sensibility or pain when hairs were plucked out by the roots. If this be correct, we should explain it by the fact, that the animal was, by division of the nerve of expression and respiration, deprived of the power of making known its suffering; and Bellingeri's erroneous inference will thus appear to be precisely the converse of that equally erroneous one first drawn by Sir C. Bell in regard to the infra-orbitar branch of the fifth.

* Meaning those actions which are performed with the consciousness of the mind, and may be interrupted by a voluntary effort—the animal motions of Prochaska.

